# Automated Discovery of Algorithms for Molecular Electronic Structure Calculations Using Physics-Informed Program Synthesis

**DOI:** 10.1021/jacs.5c22323

**Published:** 2026-03-13

**Authors:** Kyle Acheson, Rastislav Turanyi, Scott Habershon

**Affiliations:** Department of Chemistry, 2707 University of Warwick, Coventry CV4 7AL, U.K.

## Abstract

We demonstrate a physics-informed program synthesis (PIPS) approach that can be used to identify entirely new algorithms that approximate the results of single-reference electronic structure approaches like Hartree–Fock (HF) and density-functional theory (DFT)—but without *any* self-consistent field iterations at all. Our PIPS strategy exploits the fact that the eigenvectors of the Fock matrix **F** (or Kohn–Sham matrix **K**) are the same as the eigenvectors of a broad class of matrix functions, *f*(**F**). As a result, PIPS can be used to seek matrices **M** that yield the same molecular orbital coefficients as converged HF or DFT calculations. We demonstrate this approach by generating new algorithms that accurately predict total energies for a series of heterodiatomic molecules (LiCl, LiF, NaCl, NaF) and C_1_–C_4_ hydrocarbons; further simulations of C_8_–C_20_ alkane species demonstrate further transferability and efficiency of the resulting algorithms. We obtain novel algorithms that can reproduce HF or DFT energies to within 0.1 kcal/mol/atom while requiring only a single matrix-diagonalization operation, rather than an iterative self-consistent field convergence. The approach demonstrated here could be similarly applied to more complex wave function ansatze, opening an interesting optimization-based pathway to identifying accurate yet efficient algorithms for molecular quantum chemistry.

## Graphical abstract

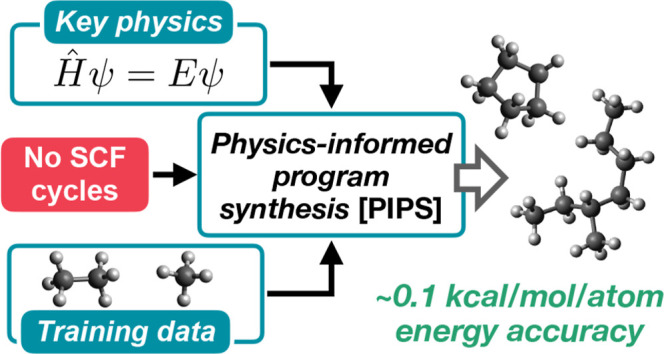

## Introduction

Artificial intelligence and machine-learning (AI/ML) approaches have, particularly over the past decade, boosted our ability to turn data and simulations into useful chemical insights. For example, the development of machine-learned atomic interaction potentials^1–6^ that are accurate, fast and transferable, has supported the exploration of new materials such as solid-state batteries for energy applications,^2^ while similar ML strategies are being developed to expand the time-scales and length-scales accessible to simulations of chemical reaction networks and nonadiabatic dynamics.^7–19^ More broadly, the rise of AI/ML—and the availability of powerful open-source software—has offered those at the interface between experimental and computational chemistry a powerful framework to extract chemical insights from complex data sets.

As an alternative to standard AI/ML strategies in the chemical sciences, symbolic regression (SR)^20–24^ has attracted some interest but is currently not at the same level of maturity, primarily due to the lower level of activity in method development and the absence of “off the shelf” software packages suited to chemical modeling. In typical SR approaches, one seeks to generate mathematical functions that accurately represent the input/output relationships contained within a given data set. In contrast to supervised AI/ML methods like artificial neural networks, where the predictive capability is encoded within a (typically large) set of floating-point weights associated with connections between nodes in different network layers, SR seeks to directly generate, assess and optimize *functions* with a definite mathematical form constructed from a set of so-called primitives, typically comprising standard mathematical operations (e.g., add, subtract, multiply, divide), floating-point parameters, and problem-specific variables. The result of SR optimization is therefore a discrete mathematical relation that aims to reproduce the expected output values given a set of training input/output pairs; compared to ANNs and similar methods, SR employs fewer free parameters, has the potential to address uneasiness around interpretability of ML models,^20,21,25^ and can result in predictive models that are simpler to represent in efficient computer codes.

Recently, we have shown how SR-type schemes can be used to generate efficient, high-accuracy, grid-based algorithms—rather than discrete functions—that solve the time-independent Schrödinger equation for bound vibrational problems.^26–28^ In this article, we show how a SR-type strategy for algorithm exploration—referred to hereafter as physics-informed program synthesis (PIPS) —can combine matrix-based nested operations with foundational approaches in quantum chemistry to yield new strategies that closely approximate the results of ab initio electronic structure methods such as Hartree–Fock (HF) theory and density functional theory (DFT). In applications to several heterodiatomic and hydrocarbon molecules, we show that PIPS can closely approximate energies from HF or DFT—but without requiring any free parameters or self-consistent field (SCF) cycles. Instead, following on from our previous work in the context of algorithm generation for solutions of the vibrational Schrödinger equation,^26–28^ we show how repeated primitive operations acting on an input workspace matrix yield good approximations to the molecular orbital coefficients (and corresponding energy expectation values) that would be calculated in traditional HF or DFT. As such, we propose that the PIPS strategy developed here can ultimately be viewed as offering a novel route to generating high-quality algorithmic approximations to matrix-based electronic structure calculations for a variety of wave function ansatze.

## Theory

We will present our PIPS approach in the context of HF theory; it is straightforward to see how the same ideas are directly applicable to Kohn–Sham DFT (and direct demonstration of this is given below and in the Supporting Information). The standard HF development leads to the set of Hartree–Fock–Roothan matrix equations^29^

1 Fc=Scϵ

where **F** is the Fock matrix evaluated in the atomic orbital (AO) basis set, **S** is the corresponding overlap matrix, **c** is the molecular orbital (MO) coefficient matrix, and **ϵ** is the set of MO energy eigenvalues. Using the standard linear-combination of atomic orbitals (LCAO) approximation, representing each one-electron MO ψ_*j*_ as a linear combination of *K* AOs ϕ_μ_

2 $$\psi_{j}=\sum_{\mu =1}^{K}c_{\mu j}\phi_{\mu }$$

the resulting Fock matrix elements are

3 $$F_{\mu \nu }=H_{\mu \nu }^{core}+\sum_{\lambda \sigma }P_{\lambda \sigma }\left[\left(\mu \nu |\sigma \lambda \right)-\frac{1}{2}\left(\mu \lambda |\sigma \nu \right)\right]$$

where *H*_μν_^core^ contains Hamiltonian matrix contributions from the one-electron kinetic energy operator and the one-electron attraction to the nuclei. The general two-electron integrals of eq 3 are defined (in atomic units) as

4 $$\left(\alpha \beta |\gamma \delta \right)=\int dr_{1}dr_{2}\frac{\phi_{\alpha }\left(1\right)\phi_{\beta }\left(1\right)\phi_{\gamma }\left(2\right)\phi_{\delta }\left(2\right)}{r_{12}}$$

and the density matrix is calculated as

5 $$P_{\mu \nu }=2\sum_{a}^{K/2}c_{\mu a}c_{\nu a}^{*}$$

where, in case of closed-shell species considered here, the summation runs over all doubly occupied MOs. The total Hartree–Fock energy can then be written in terms of AO integrals as

6 $$E_{HF}=\frac{1}{2}\sum_{\mu \nu }P_{\mu \nu }\left(H_{\mu \nu }^{core}+F_{\mu \nu }\right)$$

Given an input set of molecular geometries and corresponding energies (from HF or DFT), our goal is to use PIPS to generate new algorithms that accurately approximate these ab initio results without requiring free parameters or SCF iterations. By avoiding SCF convergence issues, we expect the resulting algorithm to operate more like a traditional force-field, while maintaining the direct connection to underlying MO theory (as well as access to MOs directly) that is often lost in AI/ML strategies for potential energy surface (PES) regression.

At first sight, we might assume that we wish to learn the functional form of a “workspace matrix” **M** as an approximation to the Fock matrix **F**, such that **M** ≃ **F**. However, as noted previously,^26–28^ the PIPS approach taken here has the particular advantage that a broad class of functions of **F** (such as those that can be defined through a Taylor series expansion) have the same *eigenvectors* as **F**. In other words, PIPS can in practice seek matrices **M** = *f*(**F**), as long as the true energy can be calculated using eq 6 (or similar). As noted previously, this gives significant flexibility in algorithms that can be discovered by PIPS, for example lending the possibility to impose sparse matrix structures for faster computation.

To achieve algorithm optimization, we use a set of “training” molecular geometries and we seek to minimize the error (typically defined by the *L* ^2^-norm) between the total energies (eq 6) predicted by HF for a given basis set, and those predicted from the MO coefficients obtained by diagonalizing the matrix **M**. This is achieved by discrete stochastic optimization of the functional form of the matrix **M**, as summarized in Figure 1.

**Figure 1:**
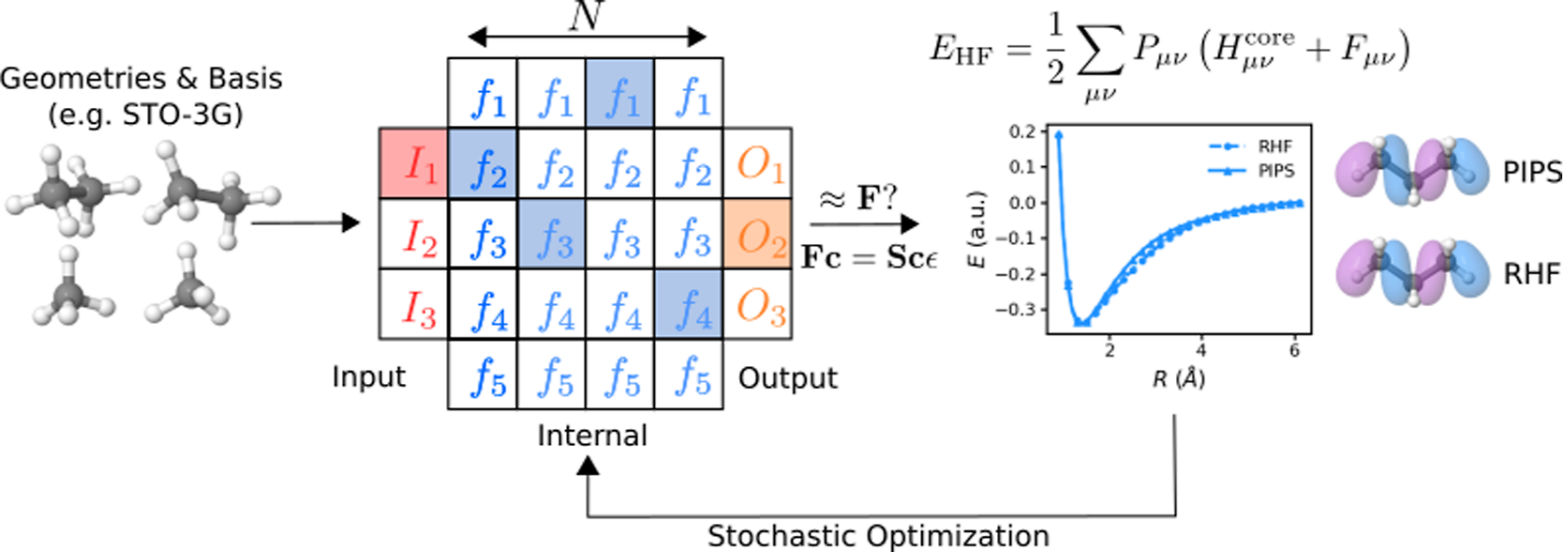
Schematic overview of our PIPS framework for electronic structure. Given a set of input geometries and a basis set, a program comprised of *N* elementary functions is stochastically optimized. This program yields an approximation to the Fock matrix **F**, which is then diagonalized to obtain coefficients **c**. Using the coefficients, the energies and MOs are subsequently predicted and compared to the ab initio reference data.

In our approach, the workspace matrix **M** is calculated using a series of functions, sequentially applied to an initial input matrix. This function search-space can be visualized as a network (Figure 1), with each column (or layer) representing application of a function onto the output matrix generated by the previous step; in other words, each “path” taken through the network corresponds to a different approximation of **M**. The network comprises input, internal-function, and output layers; the input layer functions initialize the workspace matrix (for example, using a Hückel guess), subsequent internal function-layers apply different generic mathematical operations, and—by definition—the output layer calculates the eigenvectors **c** of **M**. These eigenvectors can then be used to calculate the total HF energy (eq 6).

The optimization proceeds by first initializing a program comprising *N* operations (i.e., total layers), selected from a library of elementary functions that operate on **M** in the AO basis. In Figure 1, the colored nodes represent one possible state of the program. At each iteration, the workspace matrix approximating the Fock (or Kohn–Sham) matrix is diagonalized to yield the MO coefficients **c**. From these coefficients and the approximation to the Fock matrix, the HF energy of each geometry is computed and the fitness of the current program evaluated. A number of the *N* selected functions are then randomly updated according to the Metropolis criterion; we use a Simulated Annealing (SA) protocol with a linearly decreasing artificial temperature to drive code-performance toward lower loss-function values. Following multiple runs of PIPS on the training data, the best algorithms are selected for further testing on an extended set of molecular geometries. Crucially, as our PIPS process operates in the AO basis and yields MO coefficients, one can not only predict energies, but also the MOs themselves. Specific details of the setup of our PIPS framework, including the training/testing procedures and input/internal/output function definitions, can be found in Methods below, and in the Supporting Information.

## Methods

Here, we describe implementation of the PIPS framework outlined above, including details of training/testing protocols for heterodiatomic molecules and hydrocarbons. We focus on the description of our approach in the context of target HF calculations. Extension to Kohn–Sham DFT is straightforward; instead of seeking approximations to (functions of) the SCF-converged Fock matrix, one can similarly seek approximations to (functions of) the SCF-converged Kohn–Sham matrix, using the resulting eigenvectors in the corresponding DFT energy evaluation, as demonstrated in the Supporting Information.

### Physics-Informed Program Synthesis [PIPS]

We synthesize programs comprised of *N*_f_ elementary function operations. The total accessible function library contains 6 input matrix definitions, 183 internal functions and 2 output functions. All matrices and operations operate in the atomic orbital (AO) basis, such that the total workspace matrix size is the same as the Fock matrix, specifically *N*_AO_ × *N*_AO_ where *N*_AO_ is the number of AOs in the basis set.

Given a set of input training geometries and a basis set choice, the aim is to generate an output workspace matrix **M** that has similar eigenvectors (MO coefficients) as the SCF-converged Fock matrix **F** in eq 1. Diagonalization of **M**, followed by HF energy evaluation, should then yield energies and MOs in good agreement with the target ab initio data. We note that, while it is common to recast the Hartree–Fock–Roothan equations in eq 1 in an orthonormalized basis, we choose to work with the generalized equations for practical reasons in interfacing our PIPS optimization engine with the PySCF API used for integral and energy evaluation.^30^ The input training geometries are represented in internal coordinates, and are then internally transformed to Cartesian coordinates while fixing the molecular frame of input species consistently. This removes the need for PIPS to learn the invariance of the energies with respect to translation and rotation, while ensuring all matrix functions including AO integrals can be easily evaluated in a Cartesian basis. Similar to related deep-learning approaches,^5,6,31^ we find that attempting to learn invariance by data augmentation significantly decreases training efficiency.^32^ A more in-depth discussion of the geometry-alignment procedure can be found in the Supporting Information. As noted above, a second-generation PIPS approach incorporating rotational invariance is now an important research target.

Following transformation of the input internal coordinates to Cartesian coordinates, the PIPS procedure begins by initializing a random code of *N*_f_ functions selected from the function library. For each input training geometry and associated *N*_AO_ basis functions, a workspace matrix **M** is constructed according to the sequence of *N*_f_ selected operations. The optimization of the program then proceeds via simulated annealing (SA); at each iteration, up to three of the *N*_f_ code-functions are randomly changed, with the perturbation being accepted or rejected using the Metropolis criterion. This optimization procedures seeks to minimize the loss function given by

7 $$F_{E}=\sqrt{\frac{1}{N_{m}N_{p}}\sum_{i}^{N_{m}}\sum_{j}^{N_{p}}{\left[\left({\mathrm{E̅}}_{ij}-{\mathrm{D̅}}_{i}\right)-\left(E_{ij}-D_{i}\right)\right]}^{2}}$$

where the sum over *N*_*m*_ and *N*_*p*_ runs over the total number of different molecular species and the number of training geometries for each species, respectively. At each iteration, the workspace matrix **M** is diagonalized (eq 1), yielding the MO coefficients. The PIPS energies ${\mathrm{E̅}}_{ij}$ are subsequently predicted using eq 6.

We note that both the PIPS-predicted energies and the reference energies (*E*_*ij*_) are shifted by a characteristic constant of each molecular species. The shift factor *D*_*i*_ corresponds to the sum of atomic energies calculated using ab initio calculations (HF or DFT); in other words, energies *E*_*ij*_ are referenced to the “independent-atom” zero-of-energy. The factor ${\mathrm{D̅}}_{i}$ that shifts the PIPS-predicted energies is a free-parameter expressed as a sum of atomic contributions, which are directly optimized at each SA iteration (and are subsequently fixed in all further testing calculations). These shifts act to scale the energies of different molecules to a similar range, facilitating discovery of programs that can be applied to a variety of different molecules. As training involves identification of the atom-dependent parameter ${\mathrm{D̅}}_{i}$ , one can apply the discovered algorithms to molecules that are not included in the training data, provided that the molecules are comprised of the same set (or a subset) of elements. We note that the atomic shifts are geometry-independent; a single shift parameter for each atom-type is generated during training, with any geometry-dependence of the PES coming from the workspace matrix **M**. Using this approach, we can identify generalizable programs applicable to broad classes of molecules, as demonstrated in Figure 5. Further details regarding SA and optimization of the atomic shifts, can be found in the Supporting Information.

### Calculation Details

In the following, we outline how PIPS was used to discover approximations to functions of the Fock matrix for heterodiatomic molecules and alkanes. In both applications, we sought to reproduce the energies predicted by the RHF/STO-3G level of theory. All reference ab initio calculations were performed using the ORCA electronic structure package.^33^ We note that our choice of a minimal STO-3G basis here is driven by computational demands; the PIPS strategy is basis-set-agnostic (in the sense that PIPS can generate new algorithms that employ different basis sets when constructing the workspace matrix M; the optimized atomic shift parameters will depend on basis set, just as the independent-atom HF energies do), as demonstrated in the Supporting Information where we show that comparable accuracy can be obtained in algorithms trained on larger 6-31G basis sets. Furthermore, we note that using different basis sets for PIPS and target reference data is another algorithmic possibility; to date, we have found that the resulting algorithms obtained in this way are a little less accurate than those obtained using identical basis sets for PIPS and target data, although this mixed-basis strategy is an interesting avenue to explore in the future.

### Heterodiatomic Molecules

We ran 50 instances of PIPS on a combined training set of 45 geometries, comprised of 15 geometries of each diatomic LiCl, NaCl, and LiF. In training, we restrict sampled configurations to energies below an energy threshold *E*_max_ = 0.25 hartree (≈157 kcal/mol), ensuring a sufficient number of configurations with both small and large interatomic distances are included to capture the dissociation curve. In this case, this particular choice of energy cutoff was made because we were initially interested in whether PIPS could be used to model the potential energy surface out toward dissociation. In each PIPS instance, we used *N*_f_ = 15 operations, optimizing over *N*_iter_ = 10^4^ SA iterations starting with an initial (arbitrary) temperature of *T*_init_ = 250 × 10^3^ K. At each iteration the current code was updated by randomly modifying *r* selected functions, where *r* is a random integer generated in the range *r* ∈ [1, 3].

Following training, we studied the 15 generated codes with the lowest value of the loss function. For each of the three target training molecules, these 15 best programs were tested on their ability to reproduce the PES up to 0.8 hartree (≈502 kcal/mol) above the ground-state equilibrium energy, which includes a significant amount of internuclear Coulomb repulsion in addition to capturing dissociation. This testing set comprised 51 geometries for LiCl, 48 for NaCl, and 53 for LiF. Subsequently, we tested the best algorithms in reproducing the PES of NaF, which was not included in training data. Details of the training and testing data generation can be found in the Supporting Information. For each program, the corresponding energy shift $\overset{®}{D_{i}\;}$ for NaF was calculated using the optimized elemental contributions obtained previously during training.

### Alkanes

In the extension to polyatomic molecules, we trained 50 instances of PIPS on the two smallest hydrocarbons methane (CH_4_) and ethane (C_2_H_6_). In contrast to the heterodiatomic molecules, this required sampling the 3*N* – 6 degrees of freedom for each training molecule. An in-depth discussion of the sampling procedure can be found in Supporting Information Section 2. To summarize, we generate training points in the 3*N* – 6 dimensional space with a uniform energy distribution, reducing overfitting resulting from a biased distribution. Configurations were sampled in normal-mode coordinates, with the constraint that, in the local quadratic approximation

8 $$E=\frac{1}{2}\sum_{i}\omega_{i}^{2}q_{i}^{2}$$

all sampled energies fell below a threshold *E*_max_. For all sampled points in the set, we generated a random number ϵ ∈ [0, *E*_max_], defining the radius on the hyper-parabola as $r=\sqrt{2\epsilon }$ . Next we scale a random vector *u*, sampled on a uniform distribution, with the radius *r* and convert to normal-mode coordinates as

9 $$q_{k}=\frac{u_{k}\cdot r}{\omega_{k}}$$

where ω_*i*_ corresponding to the vibrational frequency of DOF *k*. In practice, we sampled from a subset of the total 3*N* – 6 DOF, and not the whole set collectively. While all sampled energies may be below the threshold *E*_max_ in the local quadratic approximation, this was not guaranteed for corresponding ab initio energies. So, we applied a second filtering step, removing any points where the RHF/STO-3G energies were above *E*_max_. Upon filtering of these points, the target uniform energy distribution was not guaranteed; however, by setting the threshold *E*_max_ slightly above the actual energy threshold required (and sampling sufficient points) we could achieve a uniform random distribution of energies at the RHF/STO-3G level. To generate training databases for methane and ethane, we sampled 447 and 295 points from the four lowest-frequency combinations of normal-modes, constraining the value of *E*_max_ to 0.03 hartree (≈19 kcal/mol). In this case, this choice of energy threshold was motivated by our broader interest in automated reaction discovery workflows,^34^ where we have previously investigated machine-learning models for activation energy prediction that require relative reactant/product energies as an input parameter;^35^ our hope is that methods such as PIPS could be used to calculate these relative energies.

Following sampling, we ran 50 instances of PIPS, each including 18 training points for methane and ethane (36 data-points in total). In each instance, the 18 training points were selected from the database by uniform random sampling. The same SA setup and optimization parameters outlined above for heterodiatomic molecules were also used for training against alkane data too. Upon completion of training, the best 15 PIPS runs were selected for a further two-step testing procedure. First, we tested the predictions on a set of unseen methane and ethane geometries. Crucially, the test set included geometries sampled from the four lowest-frequency modes used in training, in addition to three combinations of randomly selected higher-frequency normal-modes, summarized in Table S1 in the Supporting Information. Second, we tested the generalization of these algorithms to the next two largest hydrocarbons, propane and butane, as described in Supporting Information Section 2.2.2. In addition, we test these algorithms on selected configurations for larger alkanes, as shown in Figure 6.

## Results & Discussion

Here we focus on application of the PIPS framework to discovery of functions of the Fock operator **M** ≃ *f*(F) that provide an accurate approximation to the HF energies and MO coefficients, while avoiding the need for SCF cycles. We first demonstrate the success of PIPS-discovered algorithms in reproducing the PESs of selected heterodiatomic molecules (LiCl, LiF, NaCl, NaF), before proceeding to discuss the extension to larger molecular systems, namely C_1_–C_20_ hydrocarbons. We show that the trained algorithms can not only be accurately applied to regions of the PES outside of the training space, but they can also be applied to larger hydrocarbons not included in training, achieving subkcal/mol/atom accuracy and an *R* ^2^ > 0.99 with respect to ab initio reference data.

### Application to Heterodiatomic Molecules

We performed 50 independent optimization runs using our PIPS optimization engine to generate **M**-approximation codes with *N* = 15 total function operations, following the procedure outlined above. Each PIPS instance is trained using target ab initio data for three diatomic molecules; LiCl, NaCl, and LiF. In each case, the range of geometries and number of data points in the training set is described in the Supporting Information. The convergence of the loss function for the best-performing code at each of the *N*_iter_ = 10^4^ iterations for the 50 PIPS optimizations is shown in Figure 2a. We find that the loss function rapidly decreases within 2 × 10^3^ iterations for most runs, as is expected given that the initial codes are randomly generated. The best-performing codes typically converge with loss functions below 0.01 hartree (≈6 kcal/mol) after 8 × 10^3^ iterations, as indicated by the dashed black line. After 10^4^ iterations, the best run had an error of 5.5 kcal/mol in total energy for the training set. In the following, we implicitly assume that the training error is a guide to identifying the best-performing algorithm, but we note that performance against “hold-out” test data could be incorporated into algorithm ranking too.

**Figure 2:**
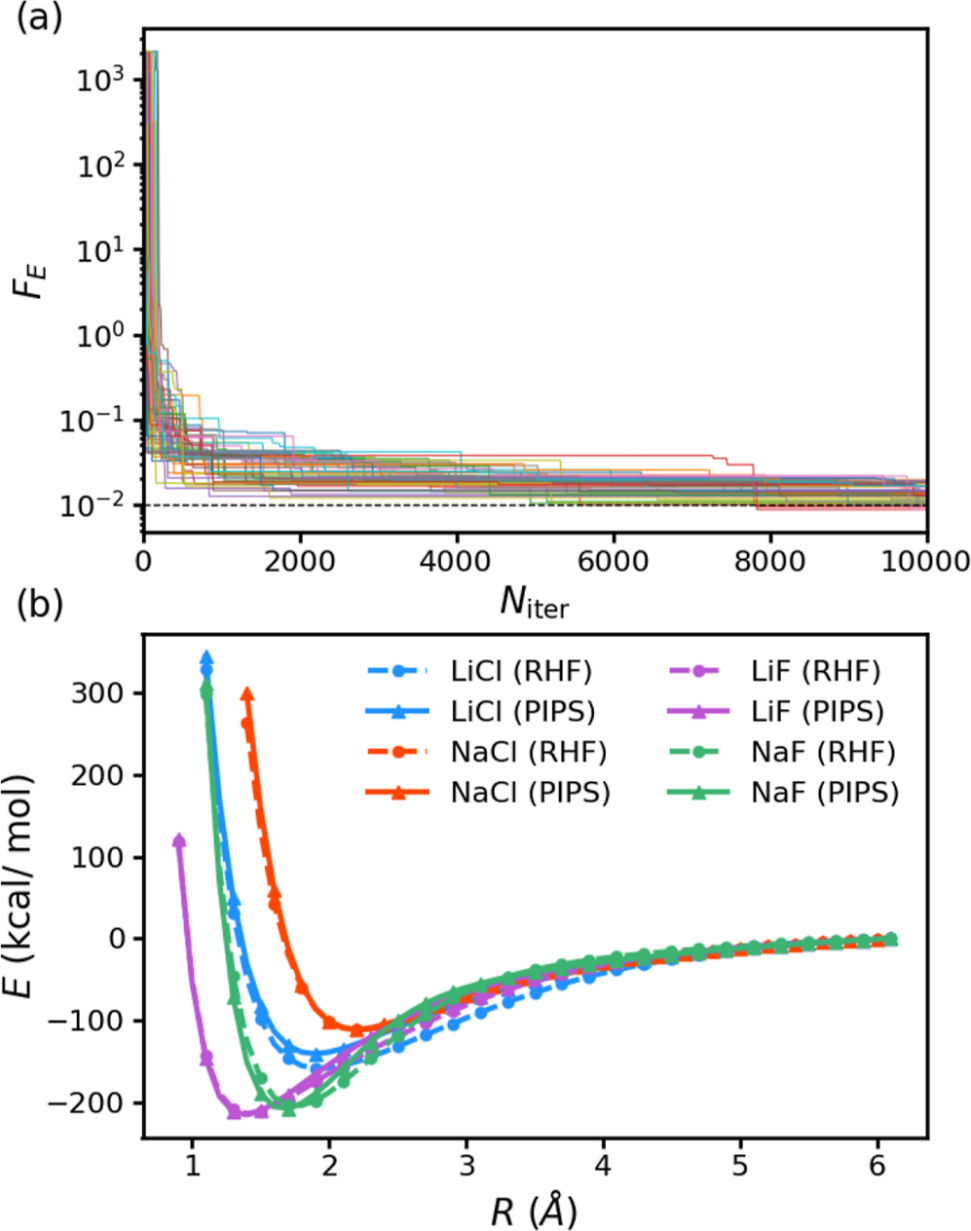
Convergence of the target for 50 runs of PIPS on a training set comprised of 15 geometries of three diatomic molecules; LiCl, NaCl, and LiF (top panel). The PIPS (algorithm **A**_1_) and RHF/STO-3G predicted PECs for the testing geometries of the three diatomics, as well as an additional NaF molecule not include in training (bottom panel). Note that here, energies are shifted such that it tends to zero at the dissociation limit. Markers are plotted for every other data point used in testing.

As an aside, we note here the difficulties in converging the target function to values lower than ≈6 kcal/mol when training multiple molecules simultaneously, particularly when including geometries corresponding to HF energies of 0.25 hartree (≈157 kcal/mol) or more above the equilibrium energy. For example, when training on a single molecular PES, we find that 30 of 50 algorithms (60%) have a training error less than 6 kcal/mol, significantly more than the four algorithms (8% success rate) identified when training for three test molecules. This finding is somewhat surprising, given that previous applications of program synthesis to solution of the time-independent vibrational Schrödinger equation found that up to 90% of PIPS runs can generate accurate algorithms.^26–28^ This comparison suggests one of several possibilities. First, it seems likely that identifying PIPS solution for the electronic Schrödinger equation is more challenging than the vibrational Schrödinger equation; in the former case, the Hamiltonian contains coupled interactions between all electrons whereas the normal-mode vibrational Hamiltonians targeted previously can be more readily approximated. Second, it is likely that the function library employed here is not optimal for the electronic structure problem. Third, it is also possible that the simple SA protocol adopted here does not support broad enough exploration of the workspace matrix-approximation landscape. All three of these points are subjects of ongoing investigation—although the proof-of-principle results demonstrated here are already promising as the basis for further development.

Next, we tested the best optimized algorithms on an independent set of geometries for each of the three heterodiatomic molecules LiCl, NaCl, and LiF. In addition, we also tested our algorithms on a hold-out molecular example that was not included in training, specifically NaF. In Figure 2b, we show the molecular PESs predicted by the best-performing algorithm (algorithm **A**_1_—see Supporting Information), which exhibited a training error of 5.5 kcal/mol. For all points within 0.8 hartree (≈502 kcal/mol) of equilibrium, the individual testing errors of LiCl, NaCl and LiF are 10.75, 7.00, and 9.33 kcal/mol, respectively. In Figure 2b, we observe that the qualitative features of the PESs are well reproduced across these three molecules. In addition, we provide plots of the same PESs with the PIPS and RHF/STO-3G curves aligned at equilibrium in Supporting Information Figures S4–S6, further highlighting that the relative features are well reproduced with most points along the curves within 5 kcal/mol of the RHF/STO-3G reference. However, we note that for LiF the largest deviations are seen around 2.0–3.5 Å, where the PIPS curve exhibits a slightly steeper slope—although the well-depth and dissociation limit are quantitatively reproduced. Furthermore, in the case of LiCl, we observe that while the curve shape is well reproduced, the well-depth/dissociation energy (depending on reference) deviate to a larger degree. Importantly, we find that the predicted relative energies for NaF—which was not included in the training data—are in good agreement with the true HF predictions, with an individual error of 10.80 kcal/mol. The corresponding plot of the NaF curves relative to equilibrium can be seen in Figure S7 of the Supporting Information. Again we observe that the qualitative features of the PES are well reproduced, although the largest deviations are seen between 2 and 3 Å, where PIPS predicts a slightly steeper slope to dissociation. The corresponding nonshifted PESs and the functional form of algorithm **A**_1_ can also be found in the Supporting Information.

To provide some further context for the observed PIPS results, we note that the order-of-magnitude fitting errors observed in these proof-of-principle calculations—with the majority of points sitting with 5 kcal/mol of the reference—is comparable to the magnitudes of dissociation energy differences associated with use of varying exchange–correlation functionals in DFT. For example, comparisons across different exchange–correlation functionals for molecules such as NaCl demonstrate variations in calculated dissociation energies of up to ≃25 kcal/mol,^36^ while a broader test of DFT exchange–correlation functions across atomization energies of 14 diatomic yields similar variations of the order of 3–25 kcal/mol.^37^

As such, we find it surprising that a computer-generated, “one-shot” algorithm can even get close to such similar error magnitudes against reference ab initio data when compared against much longer established calculation strategies. These results demonstrate that the PIPS framework can identify algorithms that can reproduce PESs of multiple heterodiatomic molecules, and provide some evidence of potential to discover algorithms that demonstrate element-level transferability, at least for the class of molecular species considered here. These results are particularly encouraging in the context of calculating dynamic properties such as vibrational spectra, where the *absolute* energies are less important than *relative* energies on the PES.

We also demonstrate here that the PIPS framework can be extended to alternative electronic structure methods, specifically DFT. Here, the HF energies within the target optimization function (eq 7) can be replaced by an analogous Kohn–Sham DFT energy evaluation. With this approach, our PIPS framework is then capable of discovering functions of the Kohn–Sham matrix **M** ≈ *f*(**K**) that similarly provide the optimized Kohn–Sham MO coefficients. In test calculations, PIPS-predicted energies for the best algorithm trained to reproduce B3LYP/STO-3G energies; as described in the Supporting Information, and employing the same training and testing details as for the HF calculations above, we find that the best algorithm can achieve training and testing errors of 5.0 and 6.2 kcal/mol, respectively. Figure 3a,c show PESs for LiCl (used in training) and NaF (absent from the training set); these results are broadly comparable to those obtained for HF target reference data, although it is noticeable that the NaF PES curve is in somewhat worse agreement when target DFT data is used. However, we also find that changes in the training protocol can impact on the performance; for example, as shown in Figure 3b and d, if one uses training-set examples with a higher energy cutoff value of 0.35 hartree (≃ 220 kcal/mol) rather than the original threshold of 0.25 hartree, the performance on the hold-out NaF examples significantly improves—possibly as a result of increased information about the broader shape of the PESs. Further details of using DFT target in PIPS are given in the Supporting Information, but the proof-of-principle results in Figure 3 highlight the potential of the PIPS strategy, and suggest that development of improved optimization schemes as an important future goal.

**Figure 3:**
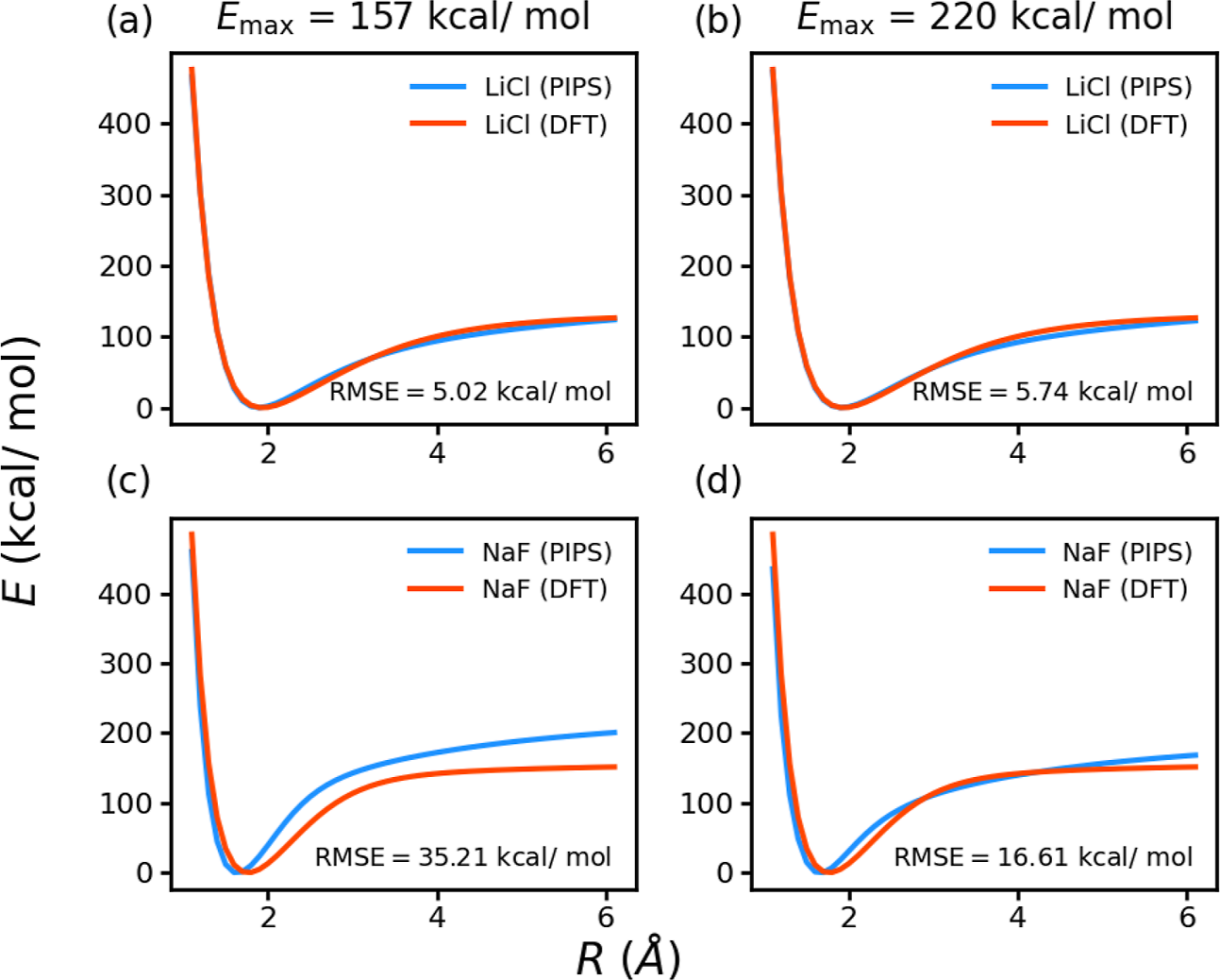
Representative results of PIPS generation using Kohn–Sham DFT reference data. Panels (a,c) show the performance of an algorithm trained against B3LYP/STO-3G data for LiF, NaCL and LiCl with a maximum energy threshold of 0.25 hartree (≃157 kcal/mol); NaF was not included in the training set. Panels (b,d) show the performance of an alternative algorithm trained on the same molecules, but with a maximum energy threshold of 0.35 hartree (≃220 kcal/mol); in this case, performance for NaF is much improved.

### Application to Alkanes

Having demonstrated that the PIPS framework can discover Fock-matrix-type approximations for heterodiatomic molecule energy evaluation, we proceeded to explore its applicability for more complex polyatomic alkane molecules. Here, a particularly important goal was to perform an initial evaluation of transferability of PIPS-generated algorithms to molecular systems that were not included in the training data. We this in mind, we first constructed a training set with 18 methane (CH_4_) configurations and 18 ethane (C_2_H_6_) configurations by uniformly sampling from the four lowest-frequency normal-mode coordinates of each molecule, as outlined in Methods above. Here, we constrained sampling to geometries lying within 0.03 hartree (≃20 kcal/mol) of the HF-predicted equilibrium configuration. As such, this proof-of-concept study aims to generate approximate HF-like algorithms that reproduce the relative energies of hydrocarbons near equilibrium, and subsequently test these algorithms for transferability to other alkane systems that were not included in the training process.

A total of 50 programs comprising *N* = 15 functions were optimized using the PIPS procedure described in Methods above. Following *N*_iter_ = 10^4^ iterations, the optimal algorithm (**A**_**2**_) was identified as having a training error of 0.37 kcal/mol. In Figure 4, we plot the PIPS-predicted energies (*E*_PIPS_) and RHF/STO-3G reference energies (*E*_RHF_) for each training geometry for each training molecule. In both cases, algorithm **A**_2_ faithfully reproduces all reference HF energies within ±1 kcal/mol, achieving *R* ^2^ > 0.99. Next, to explore potential overfitting in the optimized algorithm, we sampled further sets of geometries by introducing displacements in the four lowest-frequency normal-modes (*q*_1_ → *q*_4_) used in training, in addition to random combinations of higher-frequency normal-modes, as detailed in the Supporting Information. The optimized PIPS code was used to evaluate energies for this combined set of test geometries; the resulting PIPS-predicted and HF reference energies are plotted in Figure 4c,d. As with the training set, we observe *R* ^2^ > 0.99 for both molecules; we find that the PIPS predictions largely falling within 1 kcal/mol of the target HF energies. The functional form of the optimized algorithm **A**_2_ is given in the Supporting Information. Importantly, these results demonstrate the optimized algorithm’s ability to provide accurate predictions—both on a wider set of geometries sampled within the training region of the PES and additional regions of the PES outside of the training set. This is a particularly encouraging result, suggesting one can “discover” generalizable algorithms capturing the underlying physics of the problem at hand.

**Figure 4:**
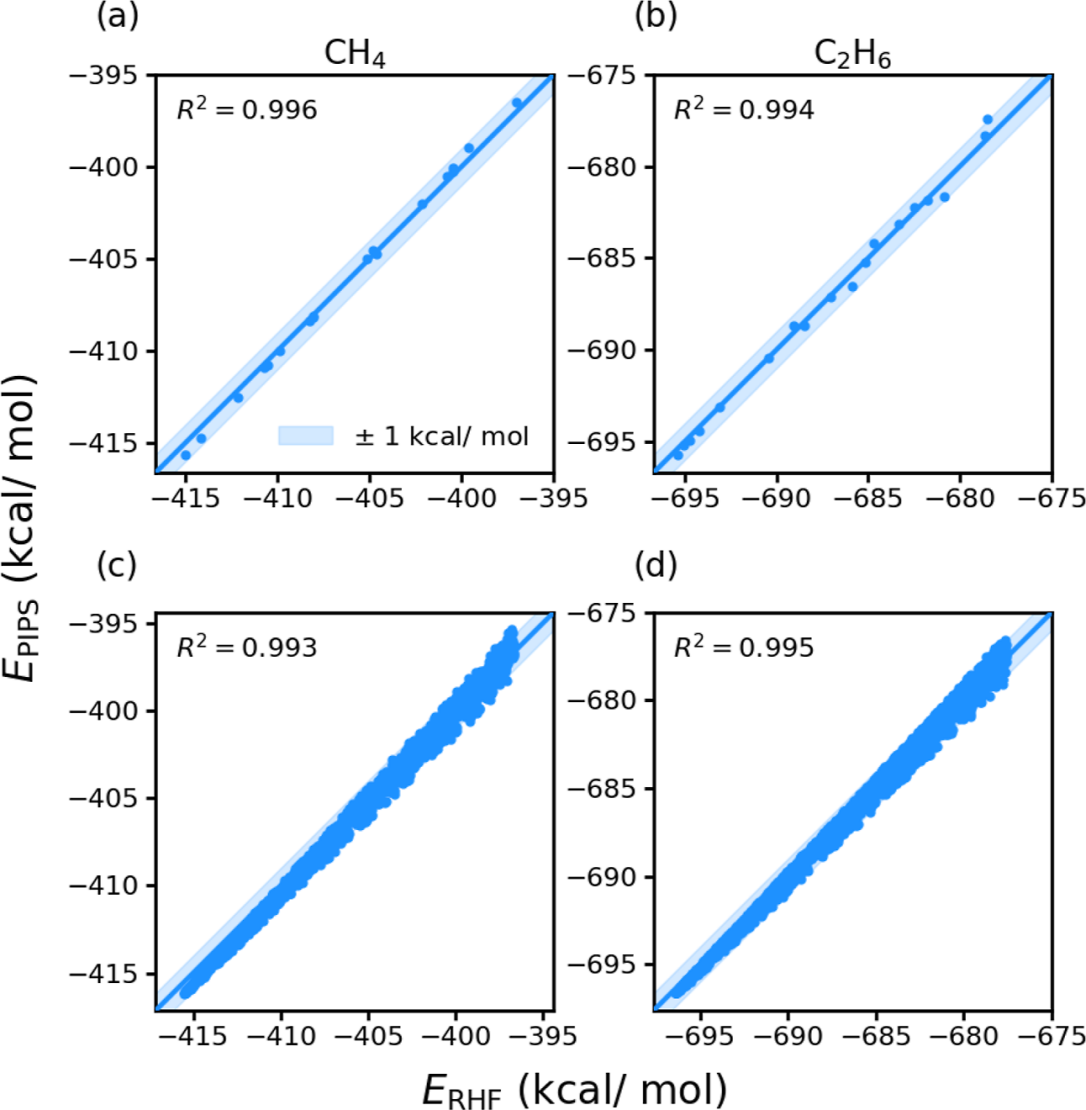
Correlation plots of algorithm **A**_2_’s PIPS predicted (*E*_PIPS_) and RHF/STO-3G reference energies (*E*_RHF_) for the two smallest hydrocarbons used in training. The training sets (top row) include 36 points sampled from the four lowest frequency normal modes. The testing sets (bottom row) include more than 2000 geometries sampled from random combinations of normal modes not used in training. In all training and testing sets, most energies are within ±1 kcal/mol of the reference RHF energies, and the value of *R* ^2^ is always >0.99.

As an aside, we note that the proof-of-principle exploration performed here results in algorithms, such as **A**_2_, that can be represented as an analytical expression but are equally somewhat difficult to “human interpret” in their current form. This is most likely a result of the inherent flexibility in the generating function *f*(**M**) noted above. However, it is straightforward to perform a simple term-importance analysis for **A**_2_ (as shown in Supporting Information Section 4.1.1), which identifies a small number of functions that can in principle be removed without dramatically degrading performance. In future work, interpretability may be further aided by introduction of “complexity constraints” into the PIPS optimization, seeking Pareto-optimal approximations that give good fits to the target data while also constraining the overall **M** complexity.

Following successful tests on the wider PES landscape of the alkane training molecules, we sought to test the limits of PIPS-optimized algorithms in generalizing to larger alkane systems that were not included in the training set. Here, we sampled geometries by randomly displacing the four lowest-frequency modes (*q*_1_ → *q*_4_) of propane (C_3_H_8_) and *n*-butane (C_4_H_10_). The correlation plots of the PIPS-predicted (algorithm **A**_2_) energies and HF energies are shown in Figure 5a,b for propane and *n*-butane, respectively. Remarkably, we find that all predicted total energies lie within ±1 kcal/mol of the HF reference total energy, again achieving *R* ^2^ > 0.99 in both cases, despite neither of these larger hydrocarbons being included in the training data set. In addition, Figure 5c,d shows the PIPS-predicted and HF frontier MOs for the ground–state equilibrium geometry of both propane and butane. Crucially, the shape of the PIPS-predicted MOs are in good agreement with those obtained from HF, as are the MO energies, even though neither of these properties were explicitly included as optimization targets. Of course, including further such MO-level information into the PIPS training protocol is an interesting avenue for future research.

**Figure 5:**
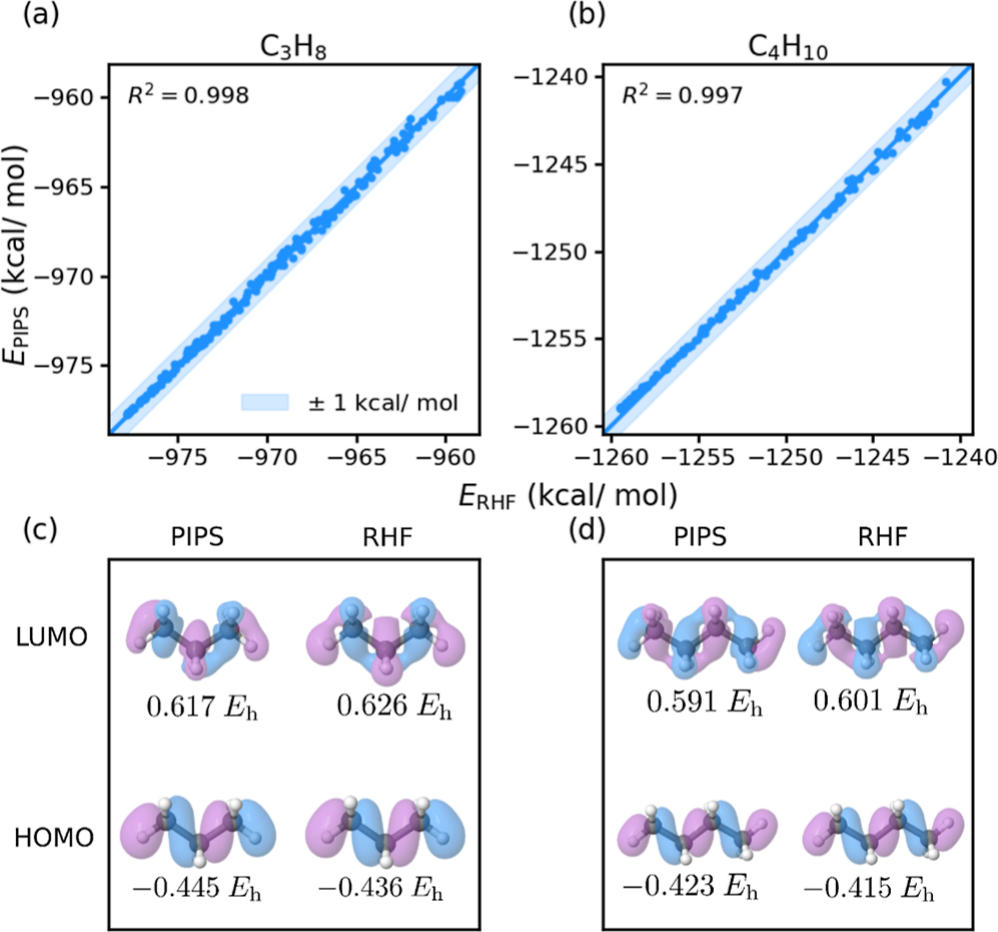
Correlation plots (top row) of algorithm **A**_2_’s PIPS predicted (*E*_PIPS_) and RHF/STO-3G reference energies (*E*_RHF_) for propane and butane, both of which are not included in training. Frontier MOs of propane and butane (bottom row) as predicted by PS and RHF for the ground state equilibrium geometry. Included for each MO is the corresponding MO energy, given in Hartree. Despite not directly training on MO energies, one can obtain MOs and MO energies that are in reasonable agreement to RHF.

So far we have focused on reproducing the PES of alkane configurations with energies up to around 19 kcal/mol, which would be broadly expected to be thermally accessible at ambient conditions. However, we now turn to demonstrating that the PIPS-generated algorithm **A**_2_ can generalize to higher-energy regions of the PES up to 60 kcal/mol above lowest-energy structures, which encompasses energies on the order of larger conformational changes. Here, we resampled configurations from the C_1_–C_4_ hydrocarbon PESs used in training and testing at three different energy thresholds, namely *E*_max_ ∈ [19, 30, 60] kcal/mol. The calculated values of the RMSE loss function over all sampled configurations are given in Table 1. Crucially, while errors increase as more higher-energy configurations are assessed, they generally remain small, with a maximum value of 1.57 kcal/mol (or ≈0.39 per/atom) for methane. This result is particularly encouraging, given that algorithm **A**_**2**_ was only trained on energies up to 0.03 hartree (≈19 kcal/mol); such results will support future exploration of novel algorithm discovery for nonequilibrium geometries such as transition states (noting the envisaged improvements in optimization and function library discussed below).

**Table 1:** Total RMSE for C_1_–C_4_ Hydrocarbons with Increasing Sampling-Energy Thresholds *E*_max_. Each Set of Points on the PES are Sampled According to the Same Procedure Employed Previously, Documented in the Supporting Information

	*F*_E_ (kcal/mol)		
molecule	*E*_max_ = 19	*E*_max_ = 30	*E*_max_ = 60
CH_4_	0.47	0.64	1.57
C_2_H_6_	0.39	0.63	1.20
C_3_H_8_	0.23	0.43	0.48
C_4_H_10_	0.27	0.40	0.41

As a further transferability test of the PIPS-generated algorithm, Figure 6 shows predicted energies obtained by PIPS compared to the corresponding HF/STO-3G calculations for five random C_8_-alkanes. In all cases, the calculated PIPS energies are within 1–3 kcal/mol of the calculated HF relative energies; the error per atom is relatively constant at around 0.1 kcal/mol/atom. Again, the PIPS algorithm used here was only optimized against a small number of configurations for CH_4_ and C_2_H_6_, so the performance for larger alkane structures (including ring-containing molecules) is a promising demonstration of transferability and performance in a low-data regime. Finally, Figure 6b shows the relative calculation times for HF/STO-3G and PIPS calculations using algorithm **A**_2_ for linear alkanes with up to 20 carbon atoms. We find that the PIPS calculation times for total energy evaluation are typically a factor of 3–5 times faster than HF/STO-3G. Furthermore, the evaluation of the molecular orbital coefficients using PIPS is even faster still, suggesting that optimization of total energy evaluation within our test code could lead to further improvements. Overall, however, this is a promising result, given that evaluation of algorithm **A**_2_ was not specifically optimized for these calculations; an interesting avenue for future work is to explore the extent to which PIPS can be modified to bias code-optimization toward algorithms that are simultaneously fast *and* accurate.

**Figure 6:**
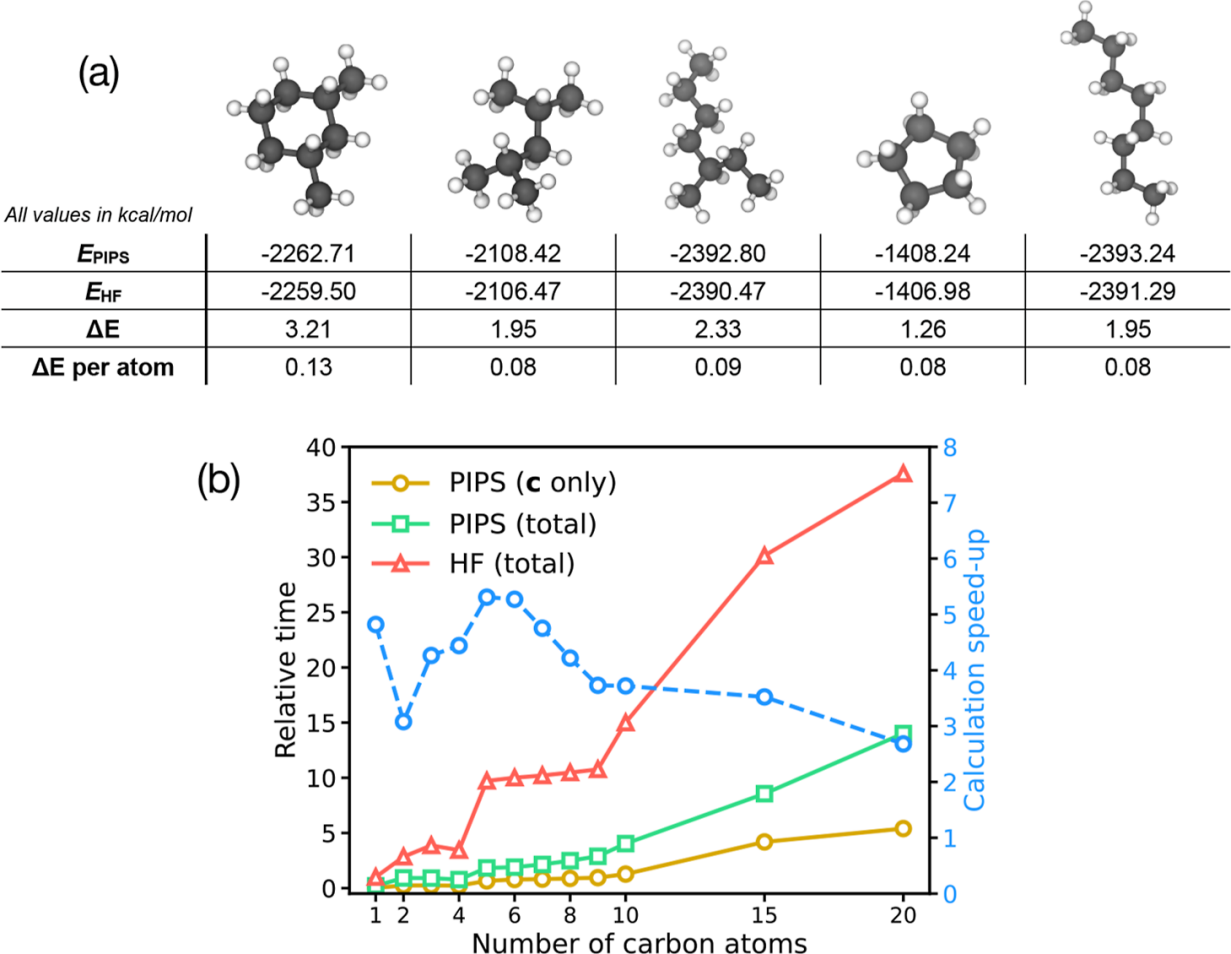
(a) Calculated energies (in kcal/mol, relative to independent atoms) and errors, Δ*E*, of five different alkane molecules, as determined by RHF/STO-3G calculations and best PIPS algorithm (**A**_2_; see Supporting Information). The PIPS code was optimized by training against CH_4_ and C_2_H_6_ data *only*, yet exhibits excellent agreement with the HF results for larger hydrocarbons. (b) Relative calculation times for energy evaluations for straight-chain alkanes with increasing number of carbon atoms. Results are shown for HF STO-3G calculations (red, triangles), and PIPS algorithm **A**_2_; for PIPS, timings are shown for both total energy evaluation (green, squares) and initial MO coefficient **c** evaluation (gold, circles). All times are shown relative to the energy evaluation time for CH_4_ using HF/STO-3G; the right–hand axis and blue dashed line shows the speed-up achieved in total energy evaluation for PIPS algorithm **A**_2_ relative to standard HF/STO-3G.

As a final speculative test of transferability of PIPS-generated algorithms, we performed calculations of the relative energies of three simple *alkene* molecules using algorithm A2—which was only trained on *alkane* geometries and energies. Somewhat surprisingly, we find that the PIPS relative energies of ethene (C_2_H_4_), propene (C_3_H_6_) and butene (C_4_H_8_) can be predicted to within 0.3–3.5 kcal/mol of the HF value at the same equilibrium geometry. Again, we find these results to be a promising indicator of potential element-level transferability on which to build in the future.

## Conclusions

To summarize, the work reported here marks a significant advance in using automated algorithm discovery approaches for quantum chemistry calculations. We have shown—for the first time—that a code-construction scheme employing discrete optimization in the space of “primitive functions” can identify new algorithms that accurately predict MOs and total energies for molecular systems, but without requiring any of the usual SCF convergence cycles that are demanded in standard HF and DFT implementations. Perhaps most interestingly, we have also demonstrated that PIPS requires only a very small amount of target training data to obtain accurate yet transferrable algorithms. For example, by training against just 36 total molecular configurations for methane and ethane, we find that the resulting PIPS-generated code is just as accurate for larger alkanes, obtaining errors *per* atom in total energies that are of the order of 0.1 kcal/mol.

Notably, the calculations performed here have also highlighted several opportunities for further improvement and development of the general PIPS strategy. First, we anticipate that expanding the function-library flexibility and training data set will improve predictive accuracy for higher-energy regions of the PES, including transition states. Second, developing new loss functions that include additional information, such as targeting reliable and accurate prediction of MO shapes and relative-energy ordering rather than total energy, is another required step that could improve PIPS performance. In addition, the potential to access atomic forces—either through direct derivation based on analytical representation of workspace matrix **M** or through automated differentiation approaches^38^—could open up further options such as including derivative information into the PIPS optimization protocol. Third, developing equivariant algorithms using PIPS—for example based on equivariant operations for construction of **M**—would be desirable, particularly in treating complex molecules and further minimizing training requirements. Finally, we note that the PIPS framework is quite general in that it could use higher-level ab initio methods, such as that from quantum Monte Carlo methods, to generate target training data; this opens the possibility of developing new “effective” algorithms that reproduce accurate quantum chemistry at lower cost. These research avenues suggest an interesting future for PIPS and related symbolic regression approaches in the context of quantum chemistry.

## Supplementary Material



## Data Availability

Data used in generating the figures is available through the Warwick Research Archive Portal at https://wrap.warwick.ac.uk/197904.

## Notes

The Supporting Information contains further details of PIPS code optimization, function library, ab initio calculations, DFT results summary, and further results relating to use of different basis sets in PIPS (PDF)
