# Supplementary material for: Automated Discovery of Algorithms for Molecular Electronic Structure Calculations Using Physics-Informed Program Synthesis
Source: J Am Chem Soc. 2026 Mar 13;148(11):11991–2000. doi: 10.1021/jacs.5c22323 (PMC13022867; doi:10.1021/jacs.5c22323)
Supplement: Supplementary file 1 [file ja5c22323_si_001.pdf]

Supporting Information: Automated discovery of algorithms for molecular electronic structure calculations using physics-informed program synthesis

Kyle Acheson, Rastislav Turanyi, and Scott Habershon*

Department of Chemistry, University of Warwick, Coventry CV4 7AL, United Kingdom

E-mail: S.Habershon@warwick.ac.uk

Contents

1 Optimization Strategy **3**

1.1 Simulated Annealing 3

1.2 Element Shift Optimization 4

2 Data Generation **6**

2.1 Procedure 6

2.2 Hydrocarbon Data 7

2.2.1 Methane and Ethane 7

2.2.2 Larger Hydrocarbons 8

2.3 Diatomic Data 9

3 Diatomic Results **11**

3.1 Restricted Hartree-Fock 11

3.1.1 STO-3G Basis 11

3.1.2 Algorithm A1 18

3.1.3 6-31G Basis 20

3.2 Density Functional Theory 26

3.2.1 Increased Energy Threshold 34

4 Hydrocarbon Results **38**

4.1 Algorithm A2: 41

4.1.1 Term-importance Analysis 41

5 Function Library **43**

Notes and references **46**

1 Optimization Strategy

1.1 Simulated Annealing

The optimization of physics-informed program synthesis (PIPS) algorithms proceeds via simulated annealing (SA), minimizing the target function,

$$F_E = \sqrt{\frac{1}{N_m N_p} \sum_{i}^{N_\mathrm{m}} \sum_{j}^{N_p} \left[\left(\bar{E}_{ij} - \bar{D}_i \right) - \left(E_{ij} - D_i \right) \right]^2}, \tag{S1}$$

with sums N_m and N_p running over the total number of different molecular species and the number of training geometries selected for each species. The energies E_{ij} are provided for by the chosen reference *ab initio* method, in this work RHF/STO-3G or B3YLP/STO-3G. The PIPS predicted energies $\bar{E}_{ij}$ are computed by diagonalization of the $N_{\mathrm{AO}} \times N_{\mathrm{AO}}$ workspace matrix $\mathbf{M}$, calculated from the N_f total selected functions at the current iteration, yielding coefficients $\mathbf{c}$. In the case of RHF reference data, the electronic part of the PIPS energy is computed from the predicted coefficients as,[1]

$$E_{\mathrm{HF}} = \frac{1}{2} \sum_{\mu\nu} P_{\mu\nu} \left(H_{\mu\nu}^{\mathrm{core}} + F_{\mu\nu} \right). \tag{S2}$$

In the case of restricted Kohn-Sham (RKS) DFT, the PIPS energy is calculated from the coefficients as,[2]

$$E_{\mathrm{KS}} = \sum_{\mu\nu} P_{\mu\nu} H_{\mu\nu}^{\mathrm{core}} + \frac{1}{2} \sum_{\mu\nu,\lambda\sigma} P_{\mu\nu} P_{\lambda\sigma} (\mu\nu|\lambda\sigma) + E_{\mathrm{xc}}[\rho], \tag{S3}$$

to both of which the nuclear repulsion E_{nuc} term is subsequently added. Following evaluation of the PIPS energies predicted by the current program state, the PIPS and *ab initio* energies are shifted by factors $\bar{D}_i$ and D_i respectively, with the former determined by an additional optimization of shift-parameters for each unique element present, as described below.

During optimization, if any numerical errors are generated by a trial algorithm - for example due to particular instabilities in $\mathbf{M}$ at some molecular geometries - this condition can be flagged

during SA to enforce rejection of the corresponding proposed algorithm.

1.2 Element Shift Optimization

The *ab initio* reference energies E_{ij} of all training geometries j of a given molecular species i are shifted by a factor D_i, constant for a given species. This shift corresponds to the sum of atomic energies,

$$D_i = \sum_{a}^{N_A} E_a, \tag{S4}$$

for each of the N_A atoms within the ith molecular species. The choice of atomic energies is a natural one, being a global and easily-computed reference for any molecular species. As such it is often employed in a variety of alternative machine-learning approaches to energy predictions. The atomic energies are computed prior to optimization, and at the same level of theory as the molecular *ab initio* energies (eg. RHF/STO-3G).

The term $\bar{D}_i$ that shifts the PIPS-predicted energies is designed to reproduce the reference shift D_i, and is computed as a sum of independent-atom terms. The shifts are determined by minimizing the sum of square residuals,

$$F_D = \sum_{i}^{N_m} \sum_{j}^{N_p} \left((\bar{E}_{ij} - \bar{D}_i) - (E_{ij} - D_i) \right)^2, \tag{S5}$$

with,

$$D_i = \sum_{u}^{N_u} c_u X_u, \tag{S6}$$

where X_u is a free-parameter for each of the N_u unique elements in the i^{ith} molecule, and c_u is a constant corresponding to the total number of each element-type in the molecule. This optimization is subject to the constraint that the parameters X_u within two summed shifts D_{i_1} and D_{i_2} are equal to each other if atom u appears in both molecules. Thus, for the whole set of training molecules, one obtains N_m total shifts $\bar{D}_i$ for each unique molecular species, obtained by summing over elemental contributions.

As an example, consider the example of training a PIPS code for hydrocarbons methane and ethane. One must then perform two optimizations for $\bar{D}_{\mathrm{CH}_4}$ and $\bar{D}_{\mathrm{C}_2\mathrm{H}_6}$, each of which are constructed as $\bar{D}_{\mathrm{CH}_4} = X_C + 4X_H$ and $\bar{D}_{\mathrm{C}_2\mathrm{H}_6} = 2X_C + 6X_H$. The values of X_C and X_H within each term $\bar{D}_{\mathrm{CH}_4}$ and $\bar{D}_{\mathrm{C}_2\mathrm{H}_6}$ are constrained to be equal, resulting in optimized values for X_C and X_H. While only optimized on methane and ethane, one can use these values to construct a corresponding shift for any hydrocarbon, allowing testing of the optimized program on molecules outside of the initial training set (as demonstrated in the main text).

The minimization of the target function in Eq. S5 is performed using the BFGS algorithm in *Scipy's minimize* function. The initial guess for each value of X_u is taken to be the atomic energy calculated using the chosen reference *ab initio* method. One may wonder why bother with this additional optimization step when the PIPS predicted energies could instead be shifted by the same value D_i as the *ab initio* reference data. The answer to this is that we aim to find broad matrix-function approximations $\mathbf{M} \approx f(\mathbf{F})/f(\mathbf{K})$ to the Fock/KS matrix that reproduce the *relative* energies of the PES. To achieve this, we make use of the fact that a general matrix may be expanded in a broad class of functions which result in the same eigenvectors but with the corresponding eigenvalues shifted by a constant amount. Thus using the same atomic shift for both the PIPS predicted and *ab initio* energies is an unnecessary constraint, enforcing the PIPS framework to attempt finding algorithms that yield the same absolute energies as the reference data. While the shift optimization procedure focuses on producing relative energies that are in reference to the independent atoms, there are more psychically meaningful alternatives such as shifting based on the dissociation or equilibrium energy. The atomic shift optimization should therefore be thought of as a simple parameterization that forces training molecules with a different energy scale to be treated on an equal footing and to avoid biasing of the target function. Importantly, this approach does not rely on including a suitable equilibrium or dissociation reference for training, and thus is completely general. Generally, we find that this procedure produces accurate relative energies across PEC's when subsequently shifted using the PIPS predicted equilibrium or dissociation energy.

2 Data Generation

2.1 Procedure

In generating training and testing for polyatomic molecules, we employ stochastic sampling of the $3N-6$ dimensional normal coordinate configuration space. To circumvent biased training on a skewed distribution, we aim to generate a uniform distribution of energies from the sampled configurations. In sampling the configuration space of a given molecule's PES calculated at a chosen level of *ab initio* theory, one first requires the corresponding optimized equilibrium geometry and Hessian to transform the sampled normal coordinates back to Cartesian coordinates.

One proceeds by selecting a maximum number of modes $N_{\max}$ to couple. Given either a predefined or randomly selected set of modes $\mathbf{q} = [q_i, q_{i_1}, \ldots, q_{N_{\max}}]$, points are sampled in a local quadratic approximation,

$$E(\mathbf{q}) = \frac{1}{2}\sum_{n=1}^{N_{\max}} \omega_{i_n}^2 q_{i_n}^2, \tag{S7}$$

subject to the constraint that the sampled energy E is below a maximum energy threshold $E_{\max}$. Note that in Eq. S7, the indices $[i_1, i_{N_{\max}}]$ refer to the nth sampled mode, and are not required to be energy ordered. To ensure that the sampled energies are guaranteed to be uniform in the range $[0, E_{\max}]$, this interval is further divided into $k_{\max} = E/\sigma$ contiguous sub-intervals,

$$I_k = [(k-1)\sigma, k\sigma) \qquad k \in [1, k_{\max} - 1], \tag{S8}$$

with the final closed sub-interval,

$$I_k = [(k-1)\sigma, E] \qquad k = k_{\max}. \tag{S9}$$

From each interval I_k, N_s configurations are sampled generating energies,

$$E_{kj} \in I_k \qquad j \in 1, \ldots, N_k. \tag{S10}$$

This is achieved by sampling N_k total random numbers $\varepsilon \in I_k$, each of which define a random radius in the N_{max} dimensional hyper-parabola as $r = \sqrt{2\varepsilon}$. Next a uniform random vector $\mathbf{u} \in \mathbb{R}^{N_{\text{max}}}$ is drawn, scaled according to the radius r, and converted to normal coordinates as,

$$q_{i_n} = \frac{u_{i_n} \cdot r}{\omega_{i_n}}. \tag{S11}$$

This is repeated for each of the N_k sampled radii r, and for each interval I_k, enforcing a uniform distribution of energies in the local quadratic approximation - providing a small enough bin spacing σ and number of samples in each bin N_k. The total number of sampled points N_s in the range $E \in [0, E_{\text{max}}]$ is then $N_k \times k_{\text{max}}$.

The normal-mode coordinate sampled points are then converted to Cartesian's through the usual transformation matrix obtained by diagonalization of the equilibrium mass-weighted Hessian. As the input to our PIPS framework is specified in internal coordinates, the Cartesian's are in turn transformed to internal coordinates, specified in Z-matrix format. As discussed in the main manuscript, the internal AO operations selected by the PIPS engine must be evaluated in Cartesian coordinates. The input internal coordinates are therefore internally transformed to Cartesian's on running PIPS. Requiring the input geometry to be specified in internal coordinates is simply a mechanism to remove the rotational and translational invariance issues. The transformation from internal to Cartesian coordinates is performed assuming that the molecular frame of all input internal coordinates is fixed consistently. Therefore, in applying any PIPS generated algorithms to configurations outside of the training set, one must ensure the molecular frame is fixed consistently over the whole set of geometries.

2.2 Hydrocarbon Data

2.2.1 Methane and Ethane

In generating methane and ethane training datasets we sample $N_k = 20$ points from $k_{\text{max}} = 19$ sub-intervals with $E_{\text{max}} = 0.03\ E_h$ and a bin width of $\sigma = 0.0016\ E_h$, resulting in a uniformly

distributed database of 380 possible training geometries (for each molecule). These training geometries are sampled by coupling the four lowest energy modes $\mathbf{q} = [q_1, \dots, q_4]$. This results in 298 (methane) and 203 (ethane) geometries with a RHF/STO-3G energy within 0.03 E_h of the ground state equilibrium energy. From this, 18 geometries from each molecular database are sampled for a given PIPS instance. The points are sampled from the wider database by binning the RHF/STO-3G energies into $k_{\max} = 3$ intervals with $E_{\max} = 0.03$ and $\sigma = 0.01$ E_h. Within each of the three intervals six random indices are drawn from a uniform distribution, giving a total of 18 selected training geometries for each molecule.

Following generation of the training databases, we turn to generating the test set. Here, we sample from random combinations of normal modes, in addition to the set of coupled modes used in training. Again the sampling procedure is conducted with $k_{\max} = 19$, $\sigma = 0.0016$ E_h, and $E_{\max} = 0.03$ E_h. The number of samples within each interval for the combination of modes (q_1, q_2, q_3, q_4) is $N_k = 30$, whereas for every other combination of modes we use $N_k = 35$. The combinations of modes sampled, along with the total number of configurations with a RHF/STO-3G energy within 0.03 E_h of equilibrium, is summarized in Tab. S1 for both methane and ethane. In testing, we assess the PIPS predictions on the combined set of all sampled geometries.

Table S1 Combinations of coupled normal modes used in sampling testing geometries for ethane and methane, including the number of sampled points N_s for each combination. In the top row, the combination of coupled modes $q_1 \to q_4$ corresponds to the sampling space used in the training phase.

CH4		C2H6	
q_i	N_s	q_i	N_s
(q_1, q_2, q_3, q_4)	447	(q_1, q_2, q_3, q_4)	295
(q_1, q_2, q_4, q_6)	530	(q_1, q_2, q_4, q_6)	382
(q_1, q_3, q_4, q_5)	527	$(q_1, q_2, q_{10}, q_{11})$	372
(q_2, q_5, q_7, q_8)	589	(q_2, q_3, q_4, q_6)	514
(q_4, q_6, q_7, q_8)	599	(q_4, q_8, q_9, q_{10})	519

2.2.2 Larger Hydrocarbons

To test the extension of our PIPS discovered algorithms to molecules outside the training set, we generate further datasets for propane and butane. In both cases, the sampling procedure is con-

ducted by coupling the four lowest frequency modes $q_1 \to q_4$, again using $k_{\max} = 19$, $\sigma = 0.0016$, and $E_{\max} = 0.03\ E_h$. This results in 205 and 141 testing geometries of propane and butane with a RHF/STO-3G energy within 0.03 E_h of equilibrium. We note that the simple sampling system we employ can struggle to effectively sample configuration-space in larger molecules; however, this is a sampling problem that is not addressed in this work. Future work should focus on exploring the vast array of sampling methods available in the literature for effectively sampling the space of larger molecules to be used in training/ testing.

2.3 Diatomic Data

Training databases comprised of LiCl, NaCl, LiF where generated such that all training geometries have an energy within 0.25 E_h of the optimized equilibrium energy. However, with only one DOF we choose not to sample geometries according to the aforementioned procedure, but instead linearly vary the corresponding bond length R_{AB}. The minimum and maximum values of R_{AB} that bound the 0.25 E_h energy threshold for each molecule are given in Tab. S2.

Table S2 Minimum and maximum values of the bond length R_{AB} varied in generating training data, values are specified in Ångström. For each molecule, we sample in the range [$R_{AB}^{\min}$, $R_{AB}^{\max}$] with N_k points, corresponding to a spacing of $\Delta R_{AB} = 0.05$ Å.

Molecule	$R_{AB}^{\min}$	$R_{AB}^{\max}$	N_k
LiCl	1.35	3.0	34
NaCl	1.6	4.0	49
LiF	1.05	3.0	40

In training our PIPS programs, for each molecule we sample a subset of $N_s = 15$ linearly spaced points from the N_k geometries in the database, with indices,

$$i_k = \left\lfloor \frac{iN_k - 1}{N_s - 1} \right\rfloor, \tag{S12}$$

resulting in 45 total training geometries across LiCl, NaCl, and LiF.

On completion of the training phase, algorithms are evaluated on a set of test geometries. This includes a hold out example not include in training, namely NaF. Here we generate geometries by

linearly varying the bond length R_{AB}, subject to the constraint that sampled geometries have an energy within 0.8 E_h of the equilibrium geometry. The values of R_{AB} sampled for each of the four molecules are summarized in Tab. S3. In contrast to the training phase where we sub-sample from the available geometries, we perform testing on the whole database of geometries.

Table S3 Minimum and maximum values of the bond length R_{AB} varied in generating testing data, values are specified in Ångström. For each molecule, we sample in the range [$R_{AB}^{\min}$, $R_{AB}^{\max}$] with N_k points, corresponding to a spacing of $\Delta R_{AB} = 0.1$ Å.

Molecule	$R_{AB}^{\min}$	$R_{AB}^{\max}$	N_k
LiCl	1.1	6.1	51
NaCl	1.5	6.1	48
LiF	0.9	6.1	53
NaF	1.1	6.1	51

3 Diatomic Results

3.1 Restricted Hartree-Fock

3.1.1 STO-3G Basis

Upon completion of 50 PIPS training runs, we identify three algorithms with a training error less than 6 kcal/ mol. In Fig. S1, we plot the histogram of optimized target function values for all runs. As discussed in the manuscript, we find it difficult to converge the target function to values lower than this when training on multiple molecular species at a time. Taking the best set of 15 algorithms from training and applying them to the wider test set of geometries, we observe only one algorithm with a testing error less than 10 kcal/mol. This algorithm ($\mathbf{A_1}$) corresponds to both the lowest training and testing errors (5.5 and 9.2 kcal/mol respectively), and is taken as the best algorithm.

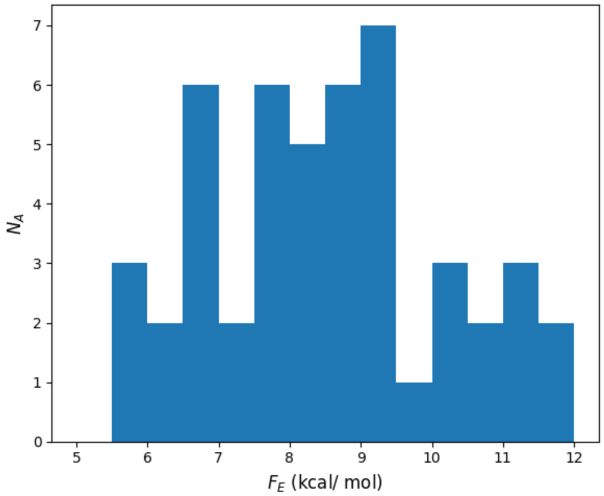

Figure S1 Histogram of final target function (F_E) values for all 50 PIPS optimization runs.

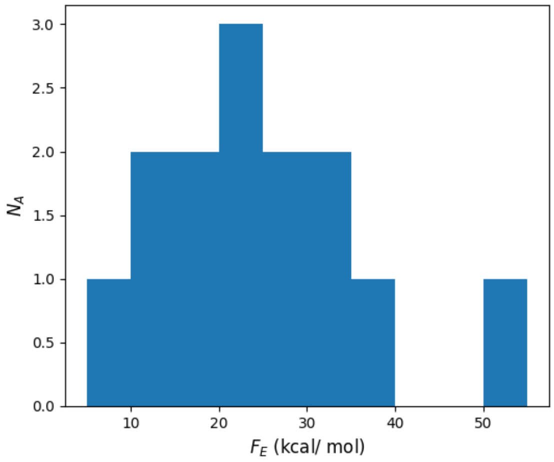

Figure S2 Histogram of target function (F_E) for the 15 algorithms with the lowest training error in Fig. S1.

In Tab. S4 we list the predicted atomic shifts resulting from the additional free-parameter optimization for algorithm $\mathbf{A_1}$, along with the RHF/STO-3G reference atomic energies. Note, the optimized free parameters faithfully reproduce the atomic RHF energies. With these parameters, one can construct a shift and apply algorithm **A1** to any molecule comprised of these atoms.

Table S4 Optimized values of the free-parameters (X_u) of algorithm $\mathbf{A_1}$, and the corresponding RHF/STO-3G calculated atomic energies ($E_{\mathrm{RHF}}^{\mathrm{atom}}$). The corresponding predicted $\bar{D}_i$ and reference D_i shift are computed by summing the respective atomic terms. All values are given in Hartree.

Atom	X_u	$E_{\mathrm{RHF}}^{\mathrm{atom}}$
Li	-7.321	-7.316
Na	-159.350	-159.668
Cl	-454.435	-454.542
F	-97.736	-97.987

In Fig. S3, we plot the PIPS and RHF/STO-3G predicted energies for the testing set of four diatomic molecules. Here both sets of energies are shifted by the sum of atomic parameters/ energies given in Tab. S4. The shifted PIPS predicted energies for all three molecules included in training (LiCl, NaCl, and LiF) are in excellent agreement with the RHF/STO-3G predictions. While the PIPS predictions for NaF (not included in training) are shifted to lower values than predicted by RHF/STO-3G, the relative features of the PEC are well reproduced, as seen in the main text where all curves are shifted so that the dissociation limit is zero.

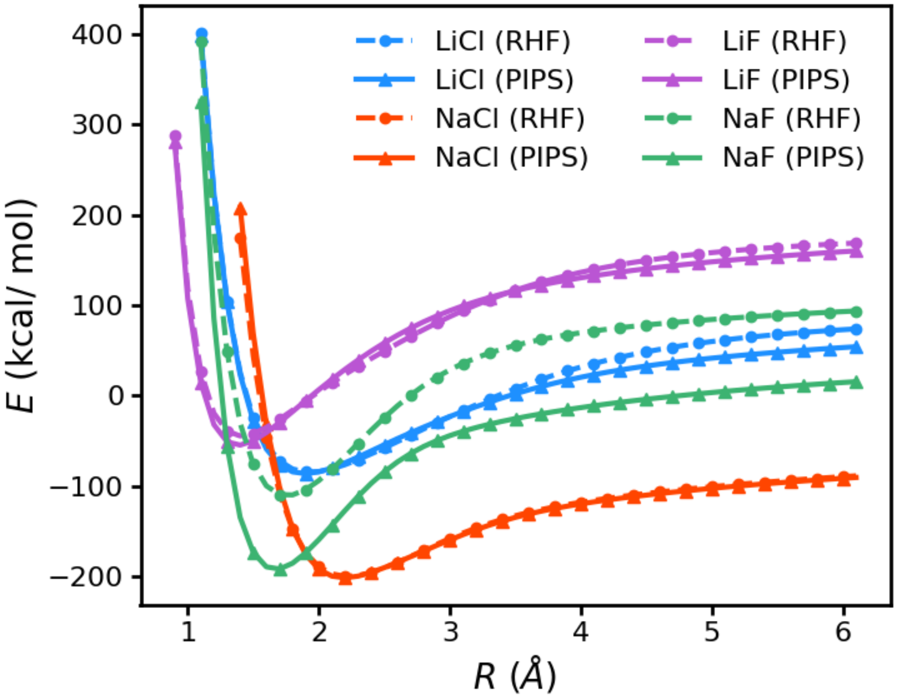

Figure S3 The PIPS and RHF/STO-3G predicted PECs for the testing geometries of the three diatomics, as well as an additional NaF molecule not include in training. Markers are plotted for every other data point used in testing. Note that here the predicted energies are shifted according to the optimized shift $\bar{D}_i$ for each molecule, and energies are not shifted so that the dissociation limit goes to zero, as in Fig. 2 of the manuscript.

In addition to the PEC's in the main text, here we show the same predictions shifted with reference to equilibrium, as seen in Fig.'s S4-S7. These figures highlight the agreement of the PIPS and RHF/STO-3G relative energies. Histograms of the differences between energy predictions also highlight where the curves are in best agreement. Crucially, it is these relative PEC's that hold the most significance. Including all sampled points up to 0.8 Hartree from equilibrium ($\approx$ 502 kcal/ mol) we observe testing errors of 10.76, 7.00, and 9.33 kcal/ mol for LiCl, NaCl, and LiF respectively. In the case of LiCl, the largest source of error is in estimating the amount of energy required for dissociation, whereas for NaCl the largest error can be seen in the repulsive region where the atoms are at an internuclear separation of around 1 Å. Finally for LiF, we note that the largest deviations are seen around 2-3.5 Åwhere the PIPS predicted PEC exhibits a slightly steeper slope towards the dissociation limit, although importantly converging to the same limit. This can also be seen in the PEC's of NaF, which was not included in training. However with a testing error of 10.80 kcal/ mol we observe that the shape and features of the PIPS predicted NaF curve are in excellent agreement with the RHF reference.

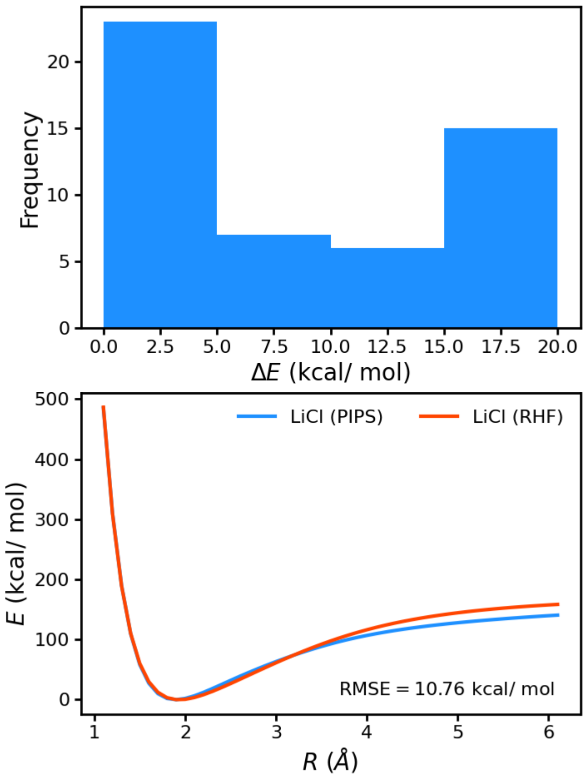

Figure S4 The PIPS and RHF/STO-3G predicted PEC for LiCl, centered on equilibrium (bottom panel), and the histogram of energy differences between curves (top panel).

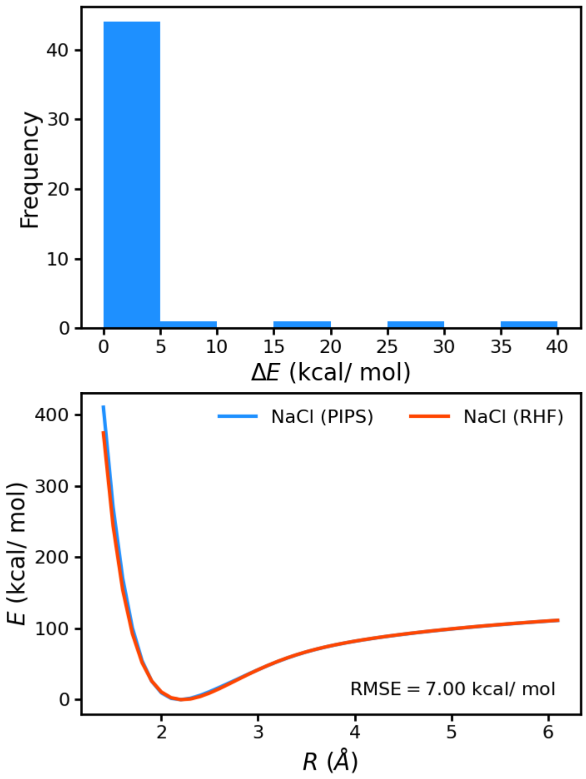

Figure S5 The PIPS and RHF/STO-3G predicted PEC for NaCl, centered on equilibrium (bottom panel), and the histogram of energy differences between curves (top panel).

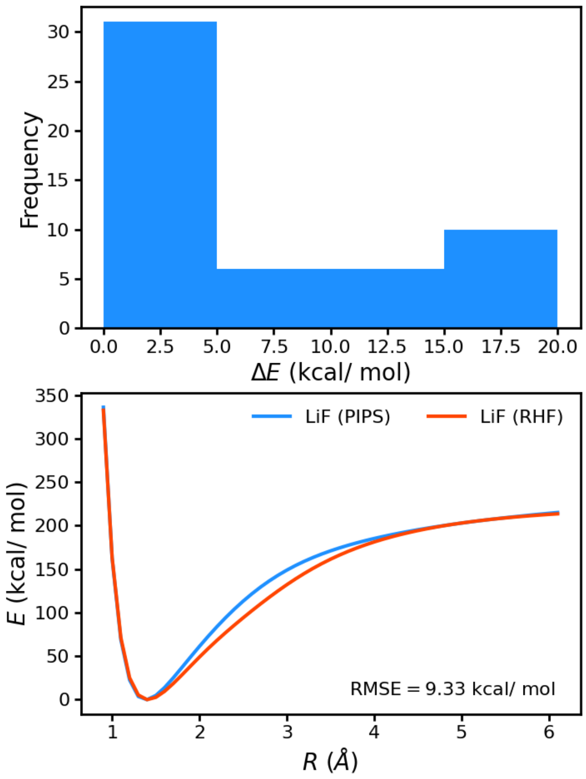

Figure S6 The PIPS and RHF/STO-3G predicted PEC for LiF, centered on equilibrium (bottom panel), and the histogram of energy differences between curves (top panel).

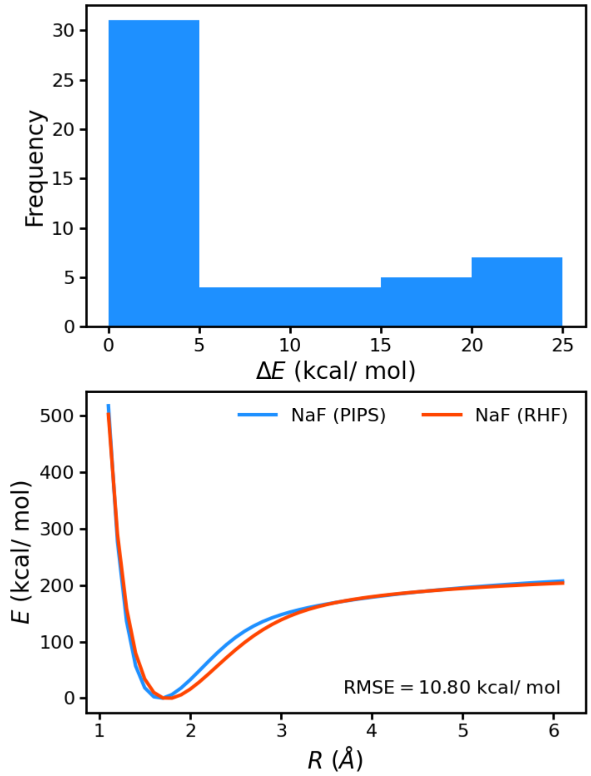

Figure S7 The PIPS and RHF/STO-3G predicted PEC for NaF, centered on equilibrium (bottom panel), and the histogram of energy differences between curves (top panel).

3.1.2 Algorithm A1

The functional form of the elements of the workspace matrix $\mathbf{M}$ are,

$$\mathbf{M} = \left[\left(\left(\exp\left(\mathbf{H}_{\text{core}} - 3\right) - \mathbf{S}^{\frac{1}{2}} \circ \mathbf{S}^{\frac{1}{2}} \right) \oslash \langle \mu\mu|\nu\nu\rangle^{-1} + 4 - \mathbf{S}^{-1} - \langle \mu\nu|\nu\nu\rangle \right) \mathbf{D} \right] \circ \langle \mu\nu|\nu\nu\rangle^{-1} \oslash \langle \mu\mu|\nu\nu\rangle \,. \tag{S13}$$

Here $\circ$ denotes element-wise multiplication (Hadamard product), and $\oslash$ element-wise division of matrix elements. All terms that appear in $\mathbf{A}_1$ are defined in Sec. 5, which details the function library. Note that this matrix is not symmetric and is subsequently symmetrized as,

$$\mathbf{M} = \mathbf{L} + \mathbf{L}^T - \text{diag}(\mathbf{L}), \tag{S14}$$

with $\mathbf{L} = \mathrm{tril}(\mathbf{M})$ the lower triangular of the workspace matrix. The PIPS predicted MO coefficients are then obtained by solving the generalized eigenvalue problem,

$$\mathbf{M}\mathbf{c} = \mathbf{S}\mathbf{c}\varepsilon, \tag{S15}$$

from which the total energy is evaluated as in Eq. S2.

3.1.3 6-31G Basis

While so far all calculations have involved the use of the minimal STO-3G basis set, we demonstrate that one can easily extend our PIPS framework to larger basis sets. To achieve this we rerun our training and testing procedures on the chosen diatomic molecules, using a PIPS framework that employs a 6-31G basis and RHF/6-31G reference energies. The corresponding PIPS and RHF predicted PECs can be seen in Fig. S8, in comparison to Fig. 2 of the main manuscript it is clear we achieve a good result with the larger 6-31G basis.

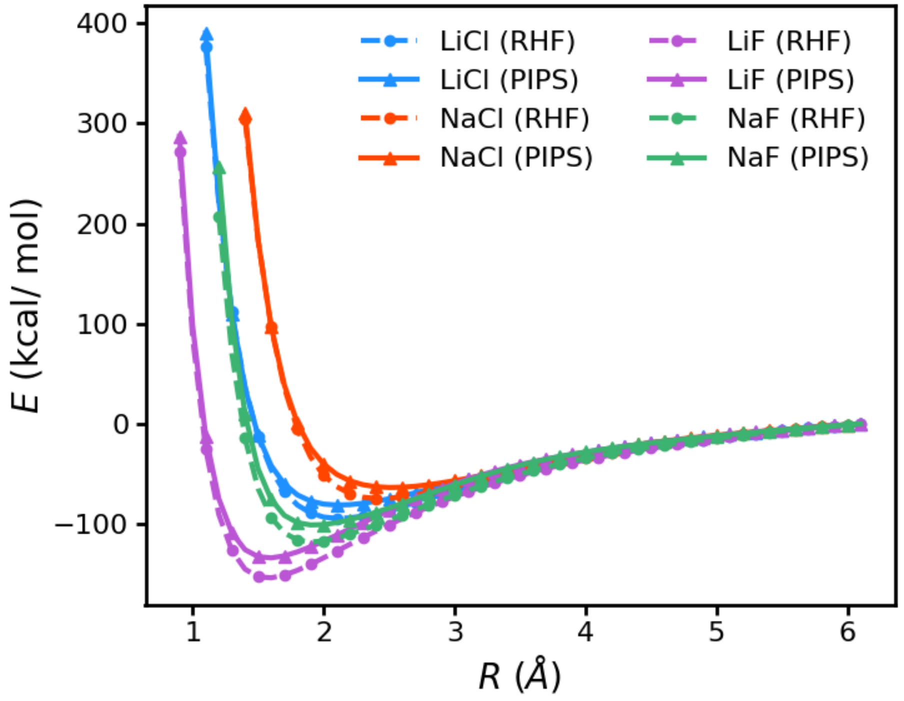

Figure S8 The PIPS and RHF/6-31G predicted PECs for the testing geometries of the three diatomics, as well as an additional NaF molecule not include in training. Markers are plotted for every other data point used in testing. Note that here the predicted energies are shifted according to the optimized shift $\bar{D}_i$ for each molecule, and the dissociation limit is shifted to zero kcal/mol.

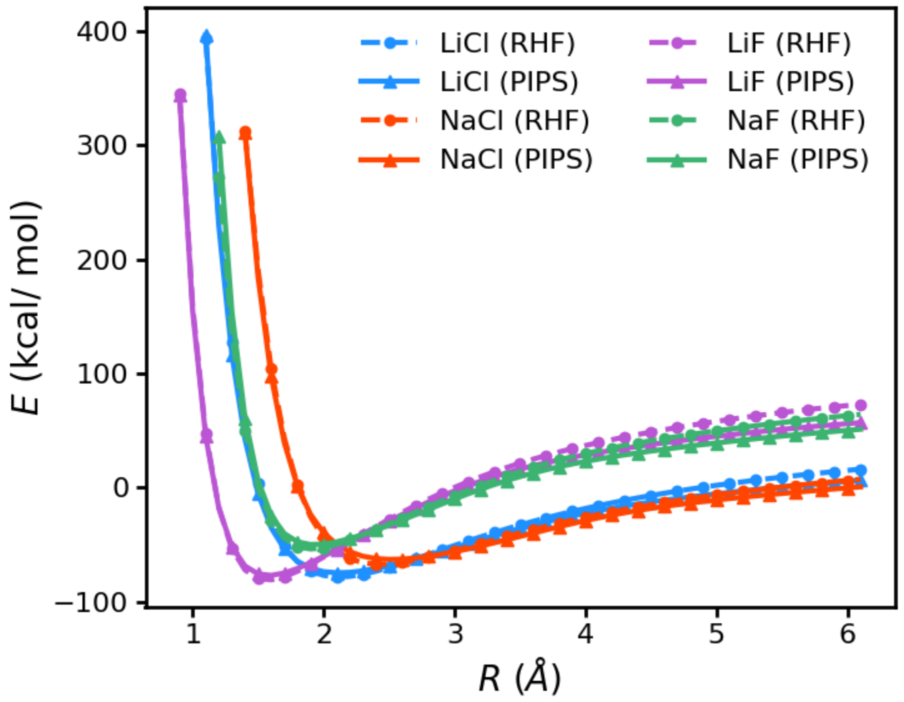

Figure S9 The PIPS and RHF/6-31G predicted PECs for the testing geometries of the three diatomics, as well as an additional NaF molecule not include in training. Markers are plotted for every other data point used in testing. Note that here the predicted energies are shifted according to the optimized shift $\bar{D}_i$ for each molecule, and the energies are not shifted so dissociation goes to zero kcal/mol, unlike Fig. S8

In addition, in Fig.'s S10-S13 we show the 6-31G PEC's shifted so that equilibrium is zero. Here we observe similar relative errors to the PIPS predictions using the smaller STO-3G basis, reproducing the shape of the PEC's of all molecules. Furthermore, as we move away from a minimal basis we observe lower dissociation energies are predicted as expected. However, this is still the largest source of error in the PIPS predicted PEC's. Here we achieve testing errors of 8.91, 7.50, 11.99, and 11.76 kcal/ mol for LiCl, NaCl, LiF, and NaF respectively.

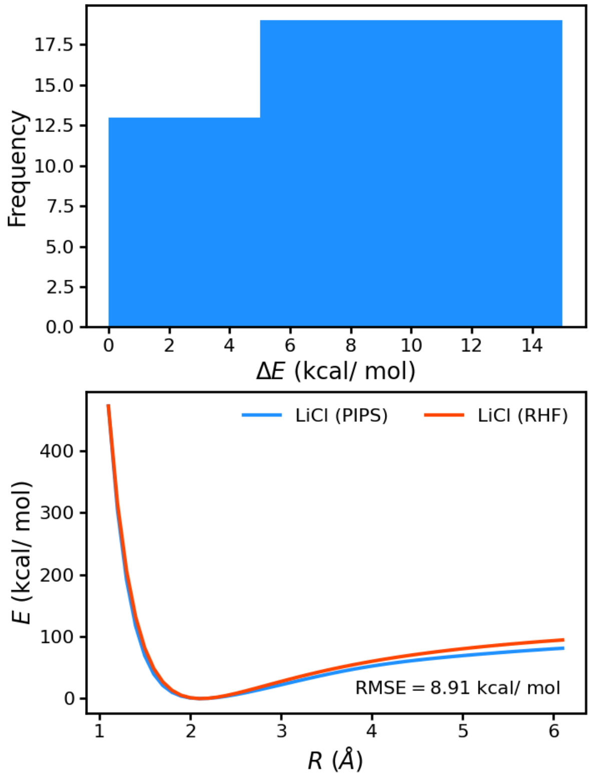

Figure S10 The PIPS and RHF/6-31G predicted PEC for LiCl, centered on equilibrium (bottom panel), and the histogram of energy differences between curves (top panel).

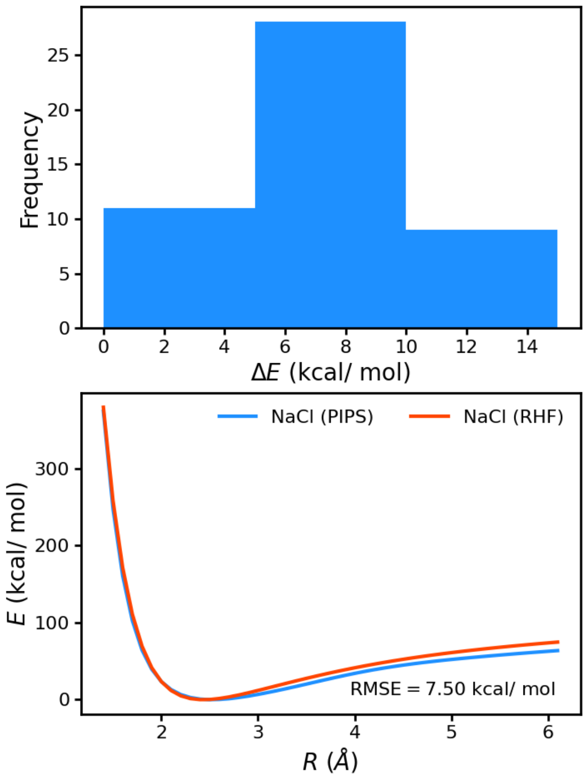

Figure S11 The PIPS and RHF/6-31G predicted PEC for NaCl, centered on equilibrium (bottom panel), and the histogram of energy differences between curves (top panel).

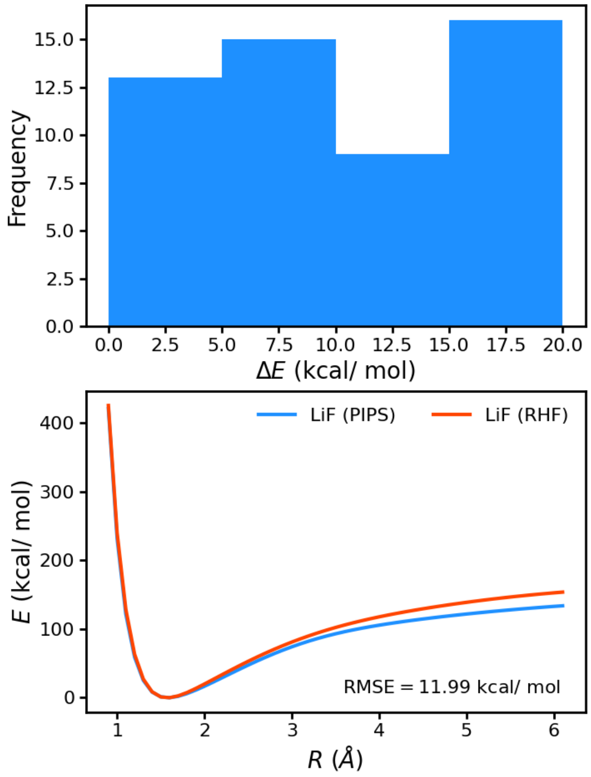

Figure S12 The PIPS and RHF/6-31G predicted PEC for LiF, centered on equilibrium (bottom panel), and the histogram of energy differences between curves (top panel).

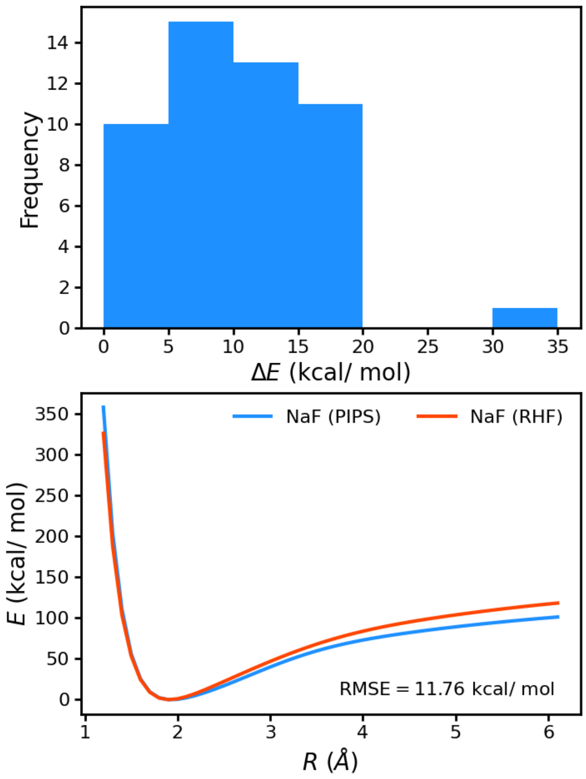

Figure S13 The PIPS and RHF/6-31G predicted PEC for NaF, centered on equilibrium (bottom panel), and the histogram of energy differences between curves (top panel).

3.2 Density Functional Theory

We test the generalization of the PIPS framework beyond simple RHF to other electronic structure methods, namely restricted Kohn-Sham (RKS) density functional theory (DFT). Here we aim to find a matrix $\mathbf{M} \approx f(\mathbf{K})$, with $\mathbf{K}$ the Kohn-Sham matrix. The energies are obtained by diagonalizing this matrix for the predicted coefficients, from which the energies are evaluated using Eq. S3 as outlined in Sec. 1 of this document. Along with redefining the energy evaluation, the only other change to the framework required is of course replacing the reference energies E_{ij} in Eq. S1 and the atomic energies in Eq. S4 with the corresponding DFT energies. We utilize the same training and testing geometries utilized in our application to RHF, the corresponding reference energies are then calculated at the B3LYP/STO-3G level of theory.

Following completion of the training phase, we identify the best PIPS algorithm with a training error of 5 kcal/ mol, comparable to the 5.5 kcal/mol training error achieved in application to RHF. The testing error for the best algorithm is found to be 6.22 kcal mol, slightly lower than the 9.20 kcal/mol testing error of RHF algorithm $\mathbf{A_1}$. In Fig. S14, we plot the PIPS and B3LYP/STO-3G energies predicted by this algorithm for the same four test molecules. Here we again shift the energies such that dissociation tends to zero kcal/mol, the corresponding unshifted plot is given in Fig. S15. Crucially, we observe that the relative energies of PECs are well reproduced. However, we note that the well-depth of the hold out example NaF is over-estimated relative to the B3LYP/STO-3G reference. While this may simply be a result of the stochastic nature of the optimization process, a more detailed analysis of PIPS performance against DFT data is planned for future work.

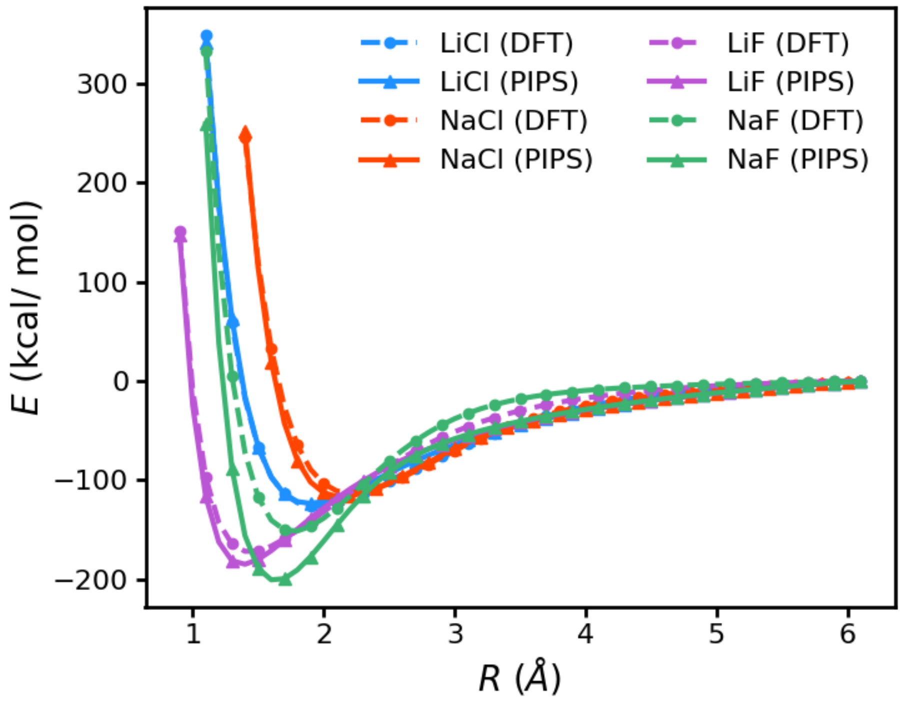

Figure S14 The PIPS and B3LYP/STO-3G predicted PECs for the testing geometries of the three diatomics, as well as an additional NaF molecule not include in training. Markers are plotted for every other data point used in testing. Note that here the energies are shifted such that dissociation goes to zero.

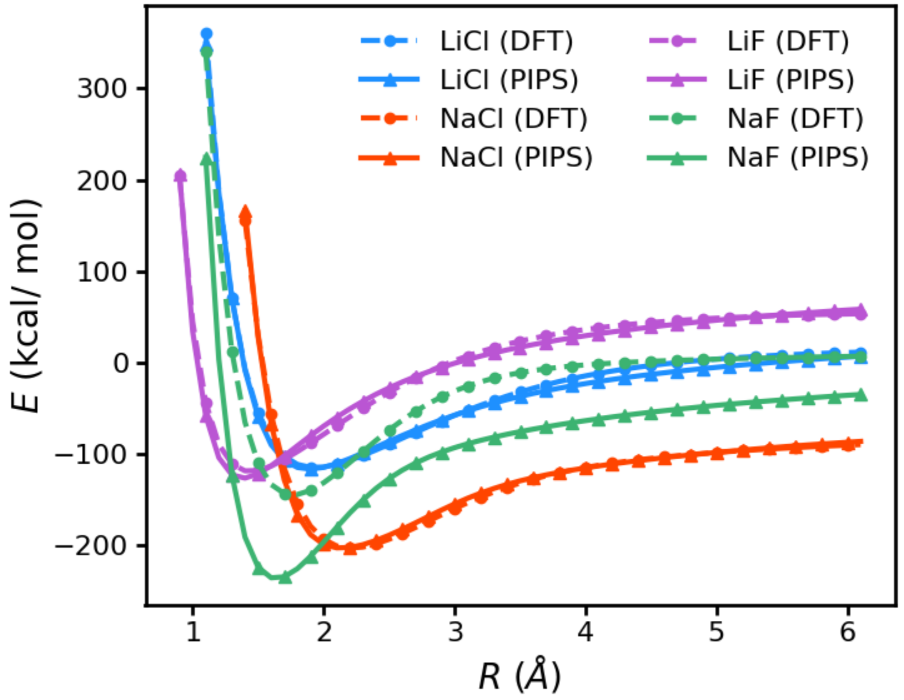

Figure S15 The PIPS and B3LYP/STO-3G predicted PECs for the testing geometries of the three diatomics, as well as an additional NaF molecule not include in training. Markers are plotted for every other data point used in testing. Note that here the predicted energies are shifted according to the optimized shift $\bar{D}_i$ for each molecule, and energies are not shifted so that the dissociation limit goes to zero, as in Fig.S14.

Table S5 Optimized values of the free-parameters (X_u) of algorithm $\mathbf{A_1}$, and the corresponding B3LYP/STO-3G calculated atomic energies ($E_{\text{DFT}}^{\text{atom}}$). The corresponding predicted $\bar{D}_i$ and reference D_i shift are computed by summing the respective atomic terms. All values are given in Hartree.

Atom	X_u	$E_{\text{DFT}}^{\text{atom}}$
Li	-7.285	-7.361
Na	-159.921	-160.041
Cl	-455.024	-455.054
F	-97.796	-98.236

The PEC's shifted so that equilibrium corresponds to zero can be seen in Fig.'s S16-S19. Generally, we again observe that the PIPS curves reproduce the shape of the B3LYP/STO-3G predictions, with both LiCl and NaCl being in excellent agreement with RMSE values of 5.02 and 4.44 kcal/ mol respectively. In the case of LiF, while the shape of the PEC is well reproduced we again note that the largest deviations are seen in the 2-3 Åregion where the PIPS curve observes a slightly larger slope, similar to the LiF RHF/STO-3G predictions in Fig. S6, although the overall error is slightly smaller. Finally, the biggest discrepancy between the PIPS and B3LYP prediction are seen for the hold out example NaF, which exhibits an overall error of 35.22 kcal/ mol and a maximum energy difference of up to 50 kcal/ mol at large internuclear separations. There exists two possible explanations of this, either it is more difficult to generalize PIPS algorithms to molecules outside the training set when using B3LYP reference data, or the stochastic optimization protocol and function library do not allow broad enough exploration of the function space that approximates the B3LYP data. Both of these issues may be addressed by ongoing research efforts. However, the generalizability of other PIPS algorithms at lower levels of theory and the reproduction of the B3LYP PEC's for molecules inside the training set are particularly encouraging results.

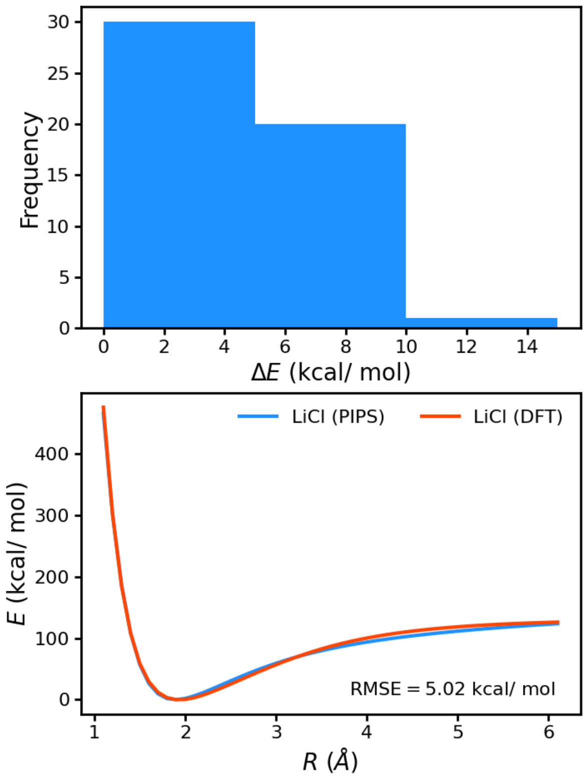

Figure S16 The PIPS and B3LYP/STO-3G predicted PEC for LiCl, centered on equilibrium (bottom panel), and the histogram of energy differences between curves (top panel).

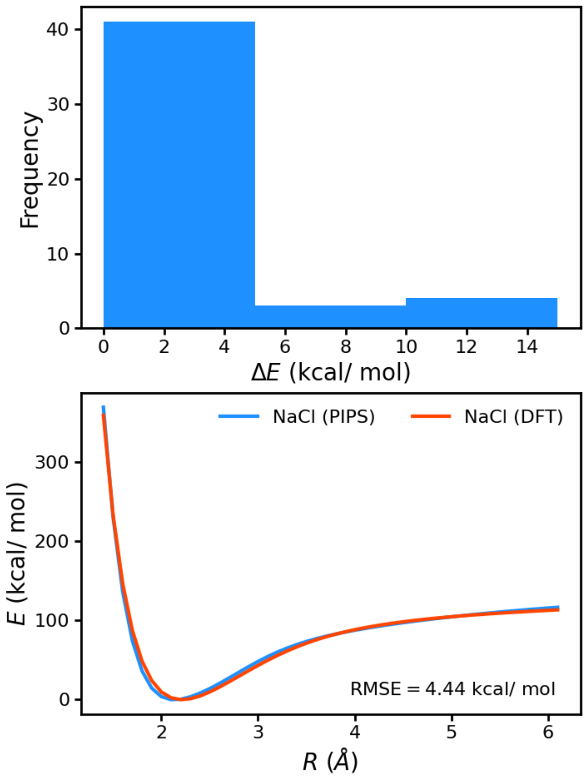

Figure S17 The PIPS and B3LYP/STO-3G predicted PEC for NaCl, centered on equilibrium (bottom panel), and the histogram of energy differences between curves (top panel).

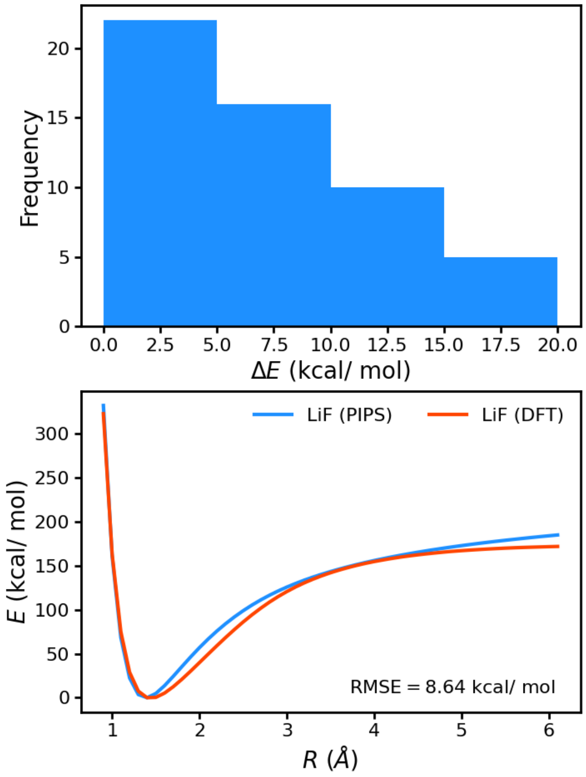

Figure S18 The PIPS and B3LYP/STO-3G predicted PEC for LiF, centered on equilibrium (bottom panel), and the histogram of energy differences between curves (top panel).

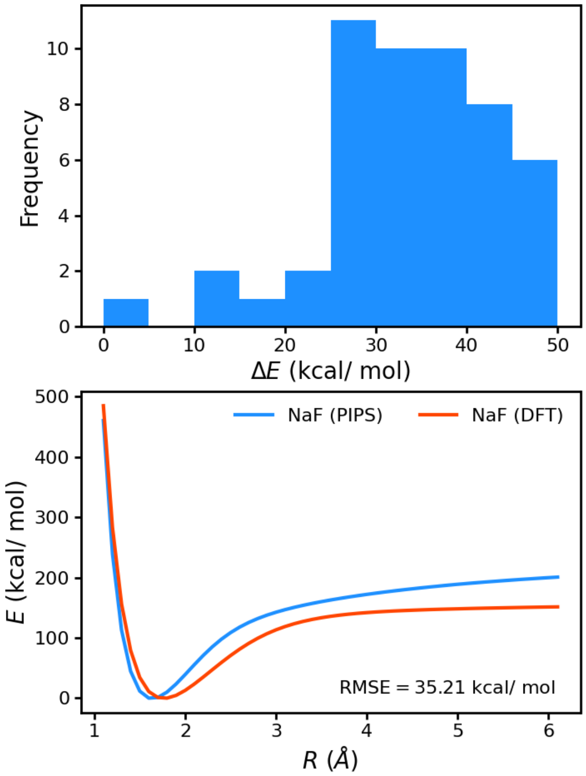

Figure S19 The PIPS and B3LYP/STO-3G predicted PEC for NaF, centered on equilibrium (bottom panel), and the histogram of energy differences between curves (top panel).

3.2.1 Increased Energy Threshold

Following the results of the previous section in which PIPS algorithms where trained on DFT energies up to 0.25 Hartree ($\approx$ 157 kcal/mol), we proceeded to retrain algorithms including energies up to 0.35 Hartree ($\approx$ 220 kcal/mol). We find that increasing the threshold slightly leads to the discovery of algorithms that improve significantly on the NaF predictions. All algorithms are again trained on the set of three testing molecules (LiCl, NaCl, LiF), the corresponding test results for each of these molecules can be seen in Figs. S20-S22. Importantly, the discovered algorithm results in an RMSE of 16.61 kcal/mol in the case of the NaF holdout example, as seen in Fig. S23. Which is a significant improvement on the 35.22 kcal/mol error observed when training with $E_{\max} = 0.25$ Hartree.

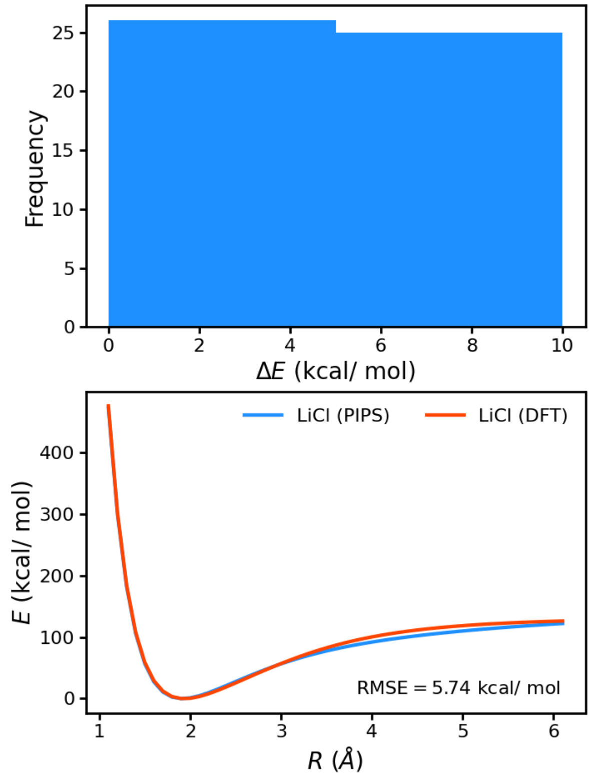

Figure S20 The PIPS and B3LYP/STO-3G predicted PEC for LiCl, centered on equilibrium (bottom panel), and the histogram of energy differences between curves (top panel). Here training is performed with $E_{\max} = 0.35$ Hartree ($\approx$ 220 kcal/mol).

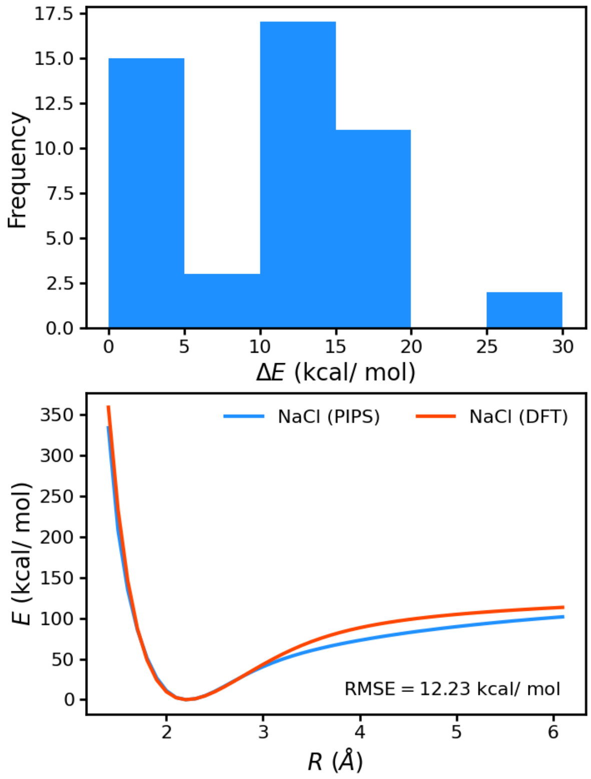

Figure S21 The PIPS and B3LYP/STO-3G predicted PEC for NaCl, centered on equilibrium (bottom panel), and the histogram of energy differences between curves (top panel). Here training is performed with $E_{\max} = 0.35$ Hartree (≈ 220 kcal/mol).

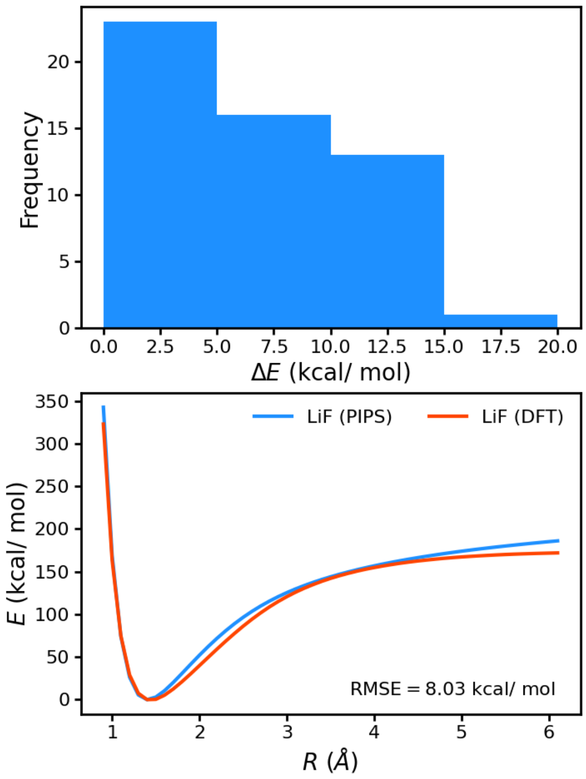

Figure S22 The PIPS and B3LYP/STO-3G predicted PEC for LiF, centered on equilibrium (bottom panel), and the histogram of energy differences between curves (top panel). Here training is performed with $E_{\max} = 0.35$ Hartree (≈ 220 kcal/mol).

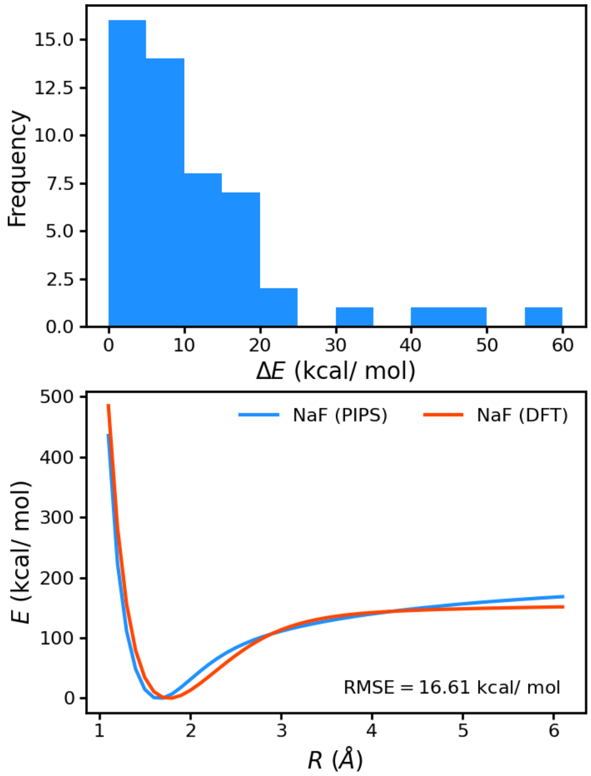

Figure S23 The PIPS and B3LYP/STO-3G predicted PEC for NaF, centered on equilibrium (bottom panel), and the histogram of energy differences between curves (top panel). Here training is performed with $E_{\text{max}} = 0.35$ Hartree (≈ 220 kcal/mol).

4 Hydrocarbon Results

Generally we achieve low errors in the range of 1-5 kcal/ mol for all 50 runs of PIPS. However, we identify two algorithms with a training error less than 1 kcal/ mol. In Fig. S24, we plot the histogram of optimized target function values for all runs. Here, the best algorithm ($\mathbf{A_2}$) has a combined training error for methane and ethane of just 0.38 kcal/ mol. Taking the best set of algorithms from training and applying them to the wider test set of methane and ethane geometries, we consistently identify algorithms with errors of less than 1 kcal/ mol. This can be seen in Fig.'s S25-S28, where we plot histograms of the testing errors for various test sets of geometries. Importantly, several algorithms achieve sub-kcal/mol accuracy for geometries sampled inside the same normal coordinate space employed in training ($q_1 \to q_4$), *and* in extended regions of normal-mode coordinate space not included in training. In each case, the best identified algorithm $\mathbf{A_2}$ always exhibits a testing error less than 1 kcal/ mol.

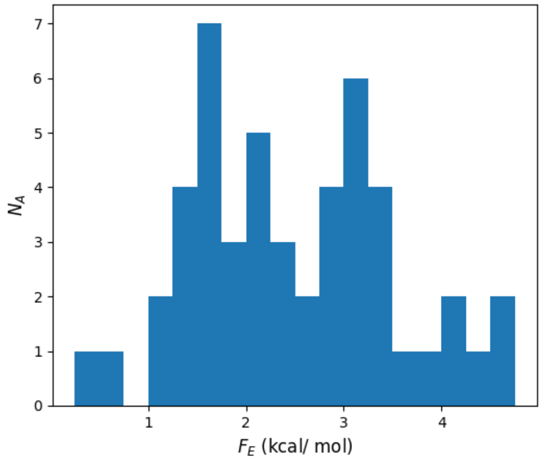

Figure S24 Histogram of final target function (F_E) values for all 50 PIPS optimization runs. Note values are combined for both methane and ethane.

In Tab. S6 we list the predicted atomic shifts resulting from the additional free-parameter optimization for algorithm $\mathbf{A_2}$, along with the RHF/STO-3G reference atomic energies. Again, the optimized free parameters faithfully reproduce the atomic RHF energies. With these optimized

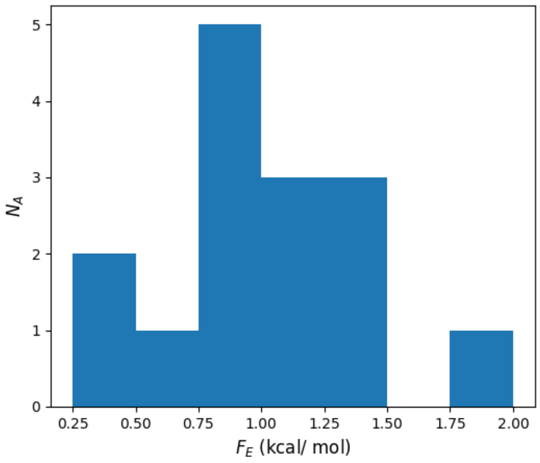

Figure S25 Histogram of target function (F_E) values of algorithms applied to the extended set of methane geometries sampled from normal modes $(q_1 \rightarrow q_4)$.

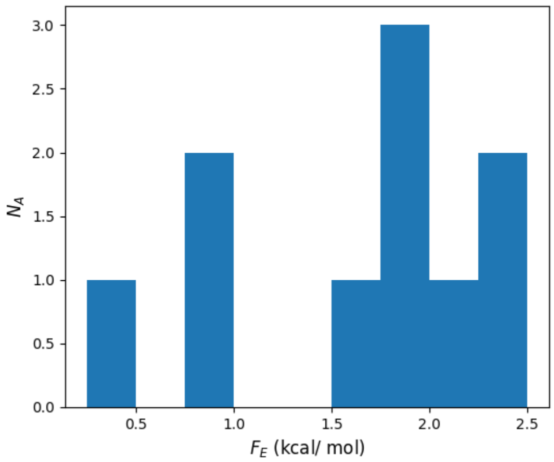

Figure S26 Histogram of target function (F_E) values of algorithms applied to the extended set of methane geometries sampled from four random combinations of normal modes not used in training (Tab. S1).

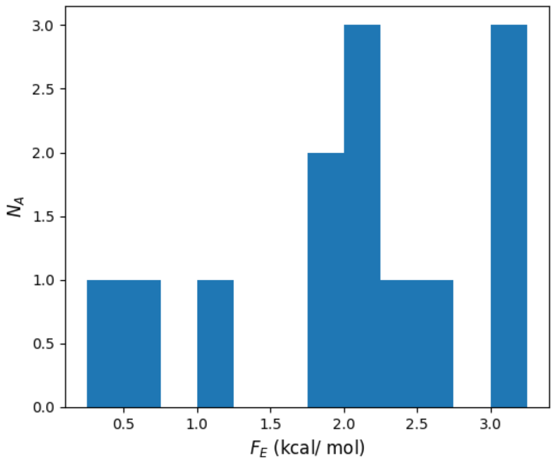

Figure S27 Histogram of target function (F_E) values of algorithms applied to the extended set of ethane geometries sampled from normal modes ($q_1 \rightarrow q_4$).

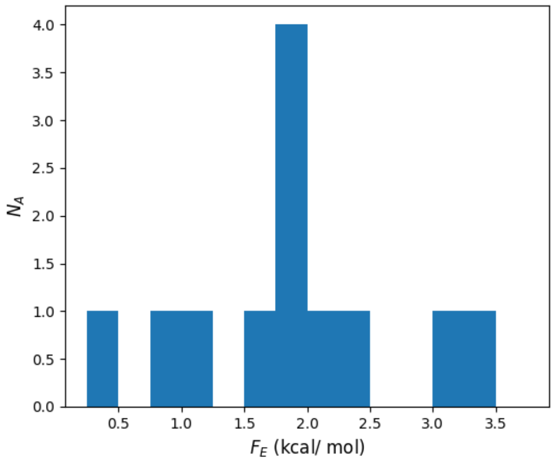

Figure S28 Histogram of target function (F_E) values of algorithms applied to the extended set of ethane geometries sampled from four random combinations of normal modes not used in training (Tab. S1).

values, algorithm $\mathbf{A_2}$ can be applied to any hydrocarbon.

Table S6 Optimized values of the free-parameters (X_u) of algorithm $\mathbf{A_2}$, and the corresponding RHF/STO-3G calculated atomic energies ($E_{\mathrm{RHF}}^{\mathrm{atom}}$). The corresponding predicted $\bar{D}_i$ and reference D_i shift are computed by summing the respective atomic terms. All values are given in Hartree.

Atom	X_u	$E_{\mathrm{RHF}}^{\mathrm{atom}}$
C	-37.199	-37.198
H	-0.465	-0.467

4.1 Algorithm A2:

The functional form of the elements of the workspace matrix $\mathbf{M}$ are,

$$\mathbf{M} = \sin\left[\left(\frac{1}{2}\left(\frac{1}{3}\mathbf{S}^{-\frac{1}{2}}\mathbf{S}\circ\langle\mu\mu|\nu\nu\rangle^{2}\right)\mathbf{T}^{2}\right)\oslash\mathbf{S}^{\frac{1}{2}}+\mathbf{S}\circ\mathbf{T}^{2}\right]\circ\mathbf{S}^{-1}\circ\langle\mu\nu|\mu\nu\rangle^{-1}\circ\mathbf{S}^{\frac{1}{2}}. \tag{S16}$$

The PIPS predicted MO coefficients are then obtained by solving the generalized eigenvalue problem,

$$\mathbf{Mc} = \mathbf{Sc}\varepsilon, \tag{S17}$$

from which the total energy is evaluated as in Eq. S2.

4.1.1 Term-importance Analysis

In analyzing the relative importance of each term in Algorithm $\mathbf{A_2}$, we remove each individual term from the equation and calculate the fractional percentage difference:

$$\Delta E_{\mathrm{term}} = 100\times|\frac{E_{\mathrm{term}}-E_{\mathrm{PIPS}}}{E_{\mathrm{PIPS}}}|, \tag{S18}$$

where E_{term} is the the energy predicted by removing the selected term and E_{PIPS} the energy predicted by algorithm $\mathbf{A_2}$ when all terms are included as usual. Fractional differences are calculated for the $C_1 - C_4$ hydrocarbons used in training and testing at their equilibrium geometry. The values of E_{term} for each hydrocarbon are given in Tab. S7, with the entries in each row corresponding to

the removal of one term. Terms are listed sequentially in order of operation, thus the form of the workspace matrix **M** in a given row is the accumulation of all preceding operations. In row one **M** corresponds to its initialized form of a matrix of ones. As operations are applied sequentially to the matrix, terms are not strictly separable. However, this provides some insight into the relative importance of the terms in the equation. For example, exclusion of terms 3, 4, 8, and 10 consistently results in the largest changes to the predicted energies across all hydrocarbons. These results highlight the importance of including non-linear terms such as powers of 2-electron integrals and trigonometric functions. Such terms are necessary for approximating the non-linear solutions of the electronic Schrödinger equation.

Table S7 Term analysis of algorithm $\mathbf{A_2}$.

Terms		ΔE_{term} (%)			
Number	Form	CH4	C2H6	C3H8	C4H10
1	$\mathbf{M} \circ S^{-\frac{1}{2}}$	6.38	8.10	7.92	9.77
2	$\mathbf{MS}$	2.75	2.35	4.21	2.61
3	$\mathbf{M} \times \frac{1}{3}$	295.52	464.11	541.10	582.11
4	$\mathbf{M} \circ \langle\mu\mu\|\nu\nu\rangle^2$	120.95	137.54	142.00	141.39
5	$\mathbf{M} \times \frac{1}{2}$	82.66	85.30	86.11	85.22
6	$\mathbf{MT}^2$	6.40	6.47	6.04	6.11
7	$\mathbf{M} \oslash \mathbf{S}^{\frac{1}{2}}$	0.79	0.91	0.62	0.51
8	$\mathbf{M} + \mathbf{S}$	3660.0	2240.30	1704.94	1417.10
9	$\mathbf{M} \circ \mathbf{T}^2$	15.23	16.91	17.35	17.81
10	$\sin(\mathbf{M})$	3743.68	4365.72	4629.18	4769.47
11	$\mathbf{M} \circ \mathbf{S}$	0.05	0.06	0.07	0.07
12	$\mathbf{M} \circ \langle\mu\nu\|\mu\nu\rangle^{-1}$	3.45	3.61	3.77	3.78
13	$\mathbf{M} \circ \mathbf{S}^{\frac{1}{2}}$	0.42	0.26	0.26	0.22

5 Function Library

In total there are 191 functions that may selected from the library, comprised of 6 input, 183 internal, and 2 output operations. All functions are specified in the following tables. In Tab. S8 we list all possible input functions that initialize the workspace matrix **M**, here functions ID's 2-3 initialize **M** as the density matrix **P** obtained from Hückel and modified Hückel guesses respectively, as detailed in Refs. 3,4. This table includes several other initialization matrices, such as the overlap matrix,

$$\mathbf{S}_{\mu\nu} = \int d\mathbf{r}_1 \phi_\mu(1)\phi_\nu(1). \tag{S19}$$

The core Hamiltonian $\mathbf{H}_{\text{core}}$ is defined as,

$$\mathbf{H}_{\text{core}} = \mathbf{T} + \mathbf{V}^{\text{nuc}}, \tag{S20}$$

with the 1-electron kinetic energy matrix,

$$\mathbf{T}_{\mu\nu} = \int d\mathbf{r}_1 \phi_\mu^*(1) \left[-\frac{1}{2}\nabla^2 \right] \phi_\nu(1), \tag{S21}$$

and the 1-electron electron-nuclear attraction matrix,

$$\mathbf{V}_{\mu\nu}^{\text{nuc}} = \int d\mathbf{r}_1 \phi_\mu^*(1) \left[-\sum_I \frac{Z_I}{|\mathbf{r} - \mathbf{R}_I|} \phi_\nu(1) \right]. \tag{S22}$$

Table S8 Table of input function operations that initialize the workspace matrix $\mathbf{M}$.

ID	operation
0	$\mathbf{M} = \mathbf{1}\mathbf{1}^T$
1	$\mathbf{M} = \mathbf{H}_{\text{core}}$
2	$\mathbf{M} = \mathbf{P}_{\text{Hückel}}$
3	$\mathbf{M} = \mathbf{P}_{\text{Hückel}}$ (modified)
4	$\mathbf{M} = \mathbf{S}$
5	$\mathbf{M} = \mathbf{I}$

Table S9 Table of internal function operations that operate on the workspace matrix $\mathbf{M}$. The possible values of $[c, \mathbf{A}, \mathbf{B}, g(), d]$ are given in Tab. S10.

ID	operation
6	null
7-10	$\mathbf{M}_{ij} \to \mathbf{M}_{ij} + c$
11-14	$\mathbf{M}_{ij} \to \mathbf{M}_{ij} - c$
15-18	$\mathbf{M}_{ij} \to \mathbf{M}_{ij} \times c$
19-22	$\mathbf{M}_{ij} \to \mathbf{M}_{ij}/c$
23	$\mathbf{M}_{ij} \to \mathbf{M}_{ij} + 1$
24	$\mathbf{M}_{ij} \to \mathbf{M}_{ij} - 1$
25	$\mathbf{M}_{ij} \to \mathbf{M}_{ij} \times -1$
26-36	$\mathbf{M}_{ij} \to \mathbf{M}_{ij} + \mathbf{A}_{ij}$
37-47	$\mathbf{M}_{ij} \to \mathbf{M}_{ij} - \mathbf{A}_{ij}$
48-58	$\mathbf{M}_{ij} \to \mathbf{M}_{ij} \times \mathbf{A}_{ij}$
59-69	$\mathbf{M}_{ij} \to \mathbf{M}_{ij}/\mathbf{A}_{ij}$
70-80	$\mathbf{M} \to \mathbf{M}\mathbf{A}$
81-91	$\mathbf{M}_{ij} \to \mathbf{M}_{ij} + \mathbf{A}^{-1}_{ij}$
92-102	$\mathbf{M}_{ij} \to \mathbf{M}_{ij} - \mathbf{A}^{-1}_{ij}$
103-113	$\mathbf{M}_{ij} \to \mathbf{M}_{ij} \times \mathbf{A}^{-1}_{ij}$
114-124	$\mathbf{M}_{ij} \to \mathbf{M}_{ij}/\mathbf{A}^{-1}_{ij}$
125-135	$\mathbf{M} \to \mathbf{M}\mathbf{A}^{-1}$
136-144	$\mathbf{M}_{ij} \to \mathbf{M}_{ij} + \mathbf{B}^{2}_{ij}$
145-153	$\mathbf{M}_{ij} \to \mathbf{M}_{ij} - \mathbf{B}^{2}_{ij}$
154-162	$\mathbf{M}_{ij} \to \mathbf{M}_{ij} \times \mathbf{B}^{2}_{ij}$
163-171	$\mathbf{M}_{ij} \to \mathbf{M}_{ij}/\mathbf{B}^{2}_{ij}$
172-180	$\mathbf{M} \to \mathbf{M}\mathbf{B}^2$
181-185	$\mathbf{M}_{ij} \to g\left(\mathbf{M}_{ij}\right)$
186-187	$\mathbf{M}_{ij} \to \mathbf{M}^{d}_{ij}$
188	$\mathbf{M}_{ij} \to \exp\left(-\mathbf{M}_{ij}\right)$

Tab. S10 includes several additional matrices which we define here. The matrix $\mathbf{D}$ is defined as the distance between atoms for each pair of atom-centered AOs,

$$\mathbf{D}_{\mu\nu} = ||R_A(\mu) - R_B(\nu)||. \tag{S23}$$

In addition, we define the following 2-electron integral matrices as slices from the rank-4 electron repulsion integral tensor,

$$\langle\mu\mu|\nu\nu\rangle = \int d\mathbf{r}_1\mathbf{r}_2 \frac{\phi_\mu(1)\phi_\mu(1)\phi_\nu(2)\phi_\nu(2)}{r_{12}}, \tag{S24}$$

$$\langle\mu\nu|\mu\nu\rangle = \int d\mathbf{r}_1\mathbf{r}_2 \frac{\phi_\mu(1)\phi_\nu(1)\phi_\mu(2)\phi_\nu(2)}{r_{12}}, \tag{S25}$$

$$\langle\mu\nu|\nu\nu\rangle = \int d\mathbf{r}_1\mathbf{r}_2 \frac{\phi_\mu(1)\phi_\nu(1)\phi_\nu(2)\phi_\nu(2)}{r_{12}}. \tag{S26}$$

The reason for including slices of the electron repulsion integral tensor is that the usual definition of the coulomb and exchange 2-electron matrices depends on the density matrix when solved in a typical SCF fashion. Here, we attempt to use PIPS to find a non-linear approximation to the MO coefficients without performing SCF cycles, therefore 2-electron terms cannot depend on contraction with the density matrix.

Table S10 Table of constants (c), matrices ($\mathbf{A}$, $\mathbf{B}$), functions (g), and powers (d) and their allowed values. These are listed in order corresponding to the function IDs in Tab. S9.

ID's	function/ argument	values
7-22	c	$[0.5, 1, 3, 4]$
26-135	$\mathbf{A}$	$[\mathbf{S}, \mathbf{T}, \mathbf{V}^{\text{nuc}}, \mathbf{S}^{-\frac{1}{2}}, \mathbf{D}, \frac{1}{\mathbf{D}}, \mathbf{S}^{\frac{1}{2}}, \langle\mu\mu\|\nu\nu\rangle, \langle\mu\nu\|\mu\nu\rangle, \langle\mu\nu\|\nu\nu\rangle, \mathbf{S}^{-1}]$
136-180	$\mathbf{B}$	$[\mathbf{D}, \frac{1}{\mathbf{D}}, \mathbf{S}, \mathbf{T}, \mathbf{V}^{\text{nuc}}, \langle\mu\mu\|\nu\nu\rangle, \langle\mu\nu\|\mu\nu\rangle, \langle\mu\nu\|\nu\nu\rangle, \mathbf{S}^{-1}]$
181-185	g	$[\exp, \sin, \cos, \ln]$
186- 187	d	$[0.5, 2]$

Table S11 Table of output function operations that diagonalize the workspace matrix $\mathbf{M}$ to yield coefficients $\mathbf{c}$ and orbital energies ε.

ID	equation to solve
189	$\mathbf{Mc} = \mathbf{Sc}\varepsilon$
190	$(\mathbf{M} + \mathbf{H}_{\text{core}})\,\mathbf{c} = \mathbf{Sc}\varepsilon$

Notes and references

(1) Szabo, A.; Ostlund, N. S. *Modern quantum chemistry: introduction to advanced electronic structure theory*; Courier Corporation, 2012.

(2) Koch, W.; Holthausen, M. C. *A Chemist's Guide to Density Functional Theory*; John Wiley Sons, Ltd, 2001.

(3) Lehtola, S. Assessment of Initial Guesses for Self-Consistent Field Calculations. Superposition of Atomic Potentials: Simple yet Efficient. *J. Chem. Theory Comput.* **2019**, *15*, 1593–1604.

(4) Ammeter, J. H.; Buergi, H. B.; Thibeault, J. C.; Hoffmann, R. Counterintuitive orbital mixing in semiempirical and ab initio molecular orbital calculations. *Journal of the American Chemical Society* **1978**, *100*, 3686–3692.
